# Integration of Position and Predictive Motion Signals in Aging Vision

**DOI:** 10.1038/s41598-020-65568-y

**Published:** 2020-05-29

**Authors:** Hyun-Jun Jeon, Yeojeong Yun, Oh-Sang Kwon

**Affiliations:** Department of Human Factors Engineering, Ulsan National Institute of Science and Technology (UNIST), Ulsan, 44919 Republic of Korea

**Keywords:** Cognitive ageing, Perception, Computational neuroscience, Sensory processing, Motion detection

## Abstract

We examined the effect of aging on the integration of position and motion signals, which is essential for tracking visual objects, using the motion-induced position shift (MIPS) phenomenon. We first measured the MIPS and bias in speed perception at three eccentricities. Both young and older adults showed the increasing MIPS and decreasing perceived speed as the eccentricity increased, which is consistent with previous literature. More importantly, we found that the mean MIPS was 2.87 times larger in older adults, and the response variability in position tasks showed a larger difference between age groups compared with the difference in speed tasks. We then measured the MIPS across stimulus durations. Temporal changes in the MIPS showed similar patterns in young and older adults in that the MIPS initially peaked at around 60 ms and approached an asymptote. We further analyzed the changes in response variability across stimulus durations to estimate sensory noise and propagation noise separately and found that only sensory noise was significantly larger in older adults. The overall results suggest that the increased MIPS in older adults is due to the increased dependency on predictive motion signals to compensate for the relatively imprecise position signals, which in turn implies that older adults would depend more on the motion signals to track objects.

## Introduction

The function of the human visual system deteriorates with aging. The ability to discriminate spatially defined features deteriorates in general. Visual acuity declines^1–6^, contrast sensitivity to high spatial-frequency stimulus is reduced^3–9^, the ability to localize the position of a peripheral object deteriorates especially when distractors are present^10^, and the ability to discriminate the shape of a configuration deteriorates^11^. A study reported that the interference of crowding stimuli increased with aging^12^, although other studies could not find consistent results^13,14^. Motion sensitivity declines also. Sensitivity to first-order (defined by luminance) and second-order (defined by other features) motion decreases as one grows older, with the second order motion processing being more vulnerable to aging^15–17^. Sensitivities to slow speed motion and speed difference are worse in older adults^18,19^, and the coherence threshold for the random dot kinematograms stimulus increases^16–18,20^. Ageing also affects the pattern-generated motion illusions such as the Rotating-Snakes and the Rotating-Tilted-Lines illusions^21^.

Declined spatial acuity and motion sensitivity in older adults are likely to weaken the ability to track a moving object because the accuracy of sensory inputs representing object position and velocity limits the accuracy of visual object tracking. The effect of aging on visual object tracking has been studied in the context of multiple object tracking. Results showed that the performances of older adults were worse, especially when the number of target objects is large^22,23^. However, multiple object tracking is not an ideal task to examine exclusively the aging effect on the process of tracking itself, because the aging effect on the task is caused by age-related changes in the ability to split attention across multiple targets^24^. It remains unanswered how the impoverished position and motion signals of older adults affect visual object tracking.

Efficient visual tracking requires more than repeatedly registering the changing positions of a moving object. The visual system has a designated sub-system for estimating the direction and speed of motion stimuli^25–27^, and the estimated direction and speed of a moving object provide predictive signals that are used to localize the position of the object^28,29^. Without the contribution of predictive motion signals, the visual system cannot track a moving object effectively^30^. Thus, it is essential to understand how the aging visual system integrates visual motion and position signals over time to grasp the effect of aging on visual object tracking. This study aimed to examine the effect of aging on the integration of position and motion signals, for which we used the motion-induced position shift (MIPS) phenomenon.

In a typical stimulus of the MIPS, a translating texture motion is presented in a stationary contrast envelope that defines an object. The perceived position of the object is systematically biased toward the direction of the texture motion when the stimulus is presented in peripheral vision^31–33^. Evidently, the MIPS is a consequence of an interaction between position and motion signals, and an elaborated account of the phenomenon is provided by a computational model which suggests that the MIPS is an output of a tracking system that optimally integrates position and motion signals^34^. According to the model, the translating texture motion is partially attributed to an object motion, and the misattributed motion signal provides predictive signals for the position estimation, which in turn biases the percept of object position. The model predicts that positional uncertainty will strongly influence the size of the MIPS. When uncertainty of the object position is high, the texture motion is more likely to be attributed to object motion and vice versa. The misattributed texture motion biases the perceived object position toward the direction of motion, while the misattributed texture motion would be lost from the perceived speed of the texture motion relative to the object. Consequently, the MIPS is expected to increase and the perceived speed of texture motion to decrease as positional uncertainty increases. Consistent with this account, the MIPS increases, and the perceived speed of texture motion generally decreases in the peripheral vision where positional uncertainty is high^34–36^.

The effect of aging on the MIPS can be influenced by three main factors: age-related changes in the sensory process of position, the sensory process of motion, or the higher-level process of integrating position and motion signals. If positional uncertainty considerably increases, assuming that the deterioration of motion sensitivity and the integration process is relatively modest, the size of the MIPS will increase. This will happen because the MIPS generally increases as positional uncertainty increases when other factors are constant^32,34^. If motion sensitivity is low or the integration process between the position and predictive motion signal is not intact while positional uncertainty is relatively low in older adults, the size of the MIPS will decrease. This will happen because intact motion perception and the influence of motion signals on position estimation are necessary for the MIPS to occur.

In Experiment 1, we measured the perceived object position and texture speed of the MIPS stimulus in older and young adults in three different eccentricities. The size of the MIPS in young and older adults allows us to examine how aging affects the degree to which motion signals modulate position estimation. By examining the changes of the MIPS and perceived texture speed in older adults as a function of eccentricity, we tested whether the pattern of the increasing MIPS and decreasing texture speed reported in previous literature for young adults is preserved in older adults. We found that the size of the MIPS was 2.87 times larger in older adults and that the effect of eccentricity was qualitatively similar in older and young adults. In Experiment 2, we measured dynamic changes in the MIPS size and response variability as a function of stimulus duration to examine how aging affects the way the visual system integrates sensory signals over time. Specifically, we were interested in testing whether older adults have a longer integration window and if the time for reaching an asymptotic level of performance is longer. The results showed that the patterns of temporal changes in the MIPS size and response variability in the two age groups were similar.

## Experiment 1

We measured the bias and response variability in the perceptual decision of position and speed of the MIPS stimuli in older and young adults to investigate the effect of aging on the integration of position and motion signals.

## Methods

### Participants

Twenty-four older adults (mean age: 72.7 ± 5.3 years, range: 61–81 years; 6 male) and twenty-one young adults (mean age: 21.7 ± 1.9 years, range: 19–25 years; 14 male) participated in the study. The sample size was determined based on the sample size of existing studies (young/old: 10/12, 16/10, 18/18) in which the effects of aging on visual tasks were examined^15,22,24^. All participants were naïve as to the purpose of the experiment. Participants self-reported that they had normal or corrected-to-normal vision and did not have visual or neurological health problems. However, it should be noted that screening participants by self-reports and performance criteria in visual tasks described below cannot fully prevent the inclusion of participants with visual and cognitive abnormalities. All procedures were approved by the Ulsan National Institute of Science and Technology institutional review board (UNISTIRB-16-14-A). All participants provided written informed consent. All methods were carried out in accordance with relevant guidelines and regulations. Seven older adults and two young participants, who did not show systematic changes in response selection as a function of stimulus change in one or more conditions, were excluded from further analysis. Systematic changes in response selection were quantified by Pearson correlation (*p* < 0.05) between response proportion (e.g., proportion of response indicating the right stimulus is higher) and stimulus property (e.g., relative height of the right stimulus). Thus, the data of 17 older adults (mean age: 71.6 ± 4.8 years, range: 61–79 years; 4 male) and 19 young adults (mean age: 21.7 ± 1.9 years, range: 19–25 years; 13 male) were analyzed. All participants were financially rewarded.

### Stimuli and apparatus

Stimuli were generated using the Matlab and Psychophysics Toolbox^37^ and presented on a DLP projector (PROPixx VPX-PRO-5050B; 1920 × 1080 resolution; 120 Hz; linear gamma). Viewing was binocular at 153 cm. The ambient and background illumination were set at 1.1 and 69.2 cd/m^2^, respectively. Fixation was enforced within a 2° fixation window at the center of the screen with an infrared eye tracker (Eyelink 1000 Plus) by aborting an ongoing trial when fixation moved out of the fixation window. A trial under the conditions of the aborted trial was added at the end of the block. A chin and forehead rest was used to minimize head movement during the experiment.

All experiments used the same base stimuli: a greyscale moving noise texture shown in a static circular contrast envelope. The noise texture was generated by applying low-pass Gaussian filters (sigma of Gaussian filter: *σ*_*f*_ = 2.1 cycles/°, isotropic in orientation) to uniform random noise^34^. Unless noted, the texture speed was 10°/s. The spatial envelope of the stimulus was a Gaussian function (i.e., a soft boundary; *σ* = 0.49°; Michelson contrast = 41.69%; RMS contrast = 6.27%), except for the reference stimulus in the speed task for which a pillbox function was used (i.e., a hard boundary; *r* = 0.65°; Michelson contrast = 42.54%; RMS contrast = 7.30%) to minimize the positional uncertainty of the reference stimulus.

### Procedure

Both young and older participants ran four blocks of the position task and four blocks of the speed task. Each block consisted of 120 trials. Older participants ran two position blocks followed by two speed blocks on the first day and repeated the same order of blocks on the second day. Young adults ran eight blocks in one day. The order of blocks was the same as for older participants. Participants were instructed to fixate on the center cross and press a button corresponding to a higher (or a faster) stimulus. After instruction, participants conducted at least 10 practice trials, which were identical to the trials in the experiments, until they were confident with the task.

**Figure 1:**
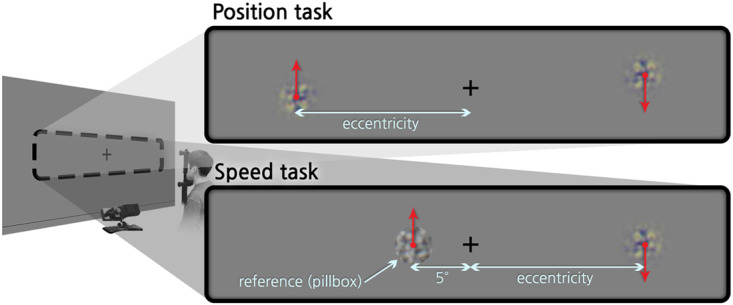
The MIPS stimuli used in position and speed tasks. In the position task, the two identical Gaussian-enveloped MIPS stimuli were presented on both sides of the screen at one of the three eccentricities, namely, 5°, 10°, and 15°. The relative vertical positions of the two stimuli were differed to PSE at which the two stimuli appear to be aligned horizontally. In the speed task, the test stimulus was presented at one of the three eccentricities, namely, 5°, 10°, and 15°. The eccentricity of the reference stimulus was fixed to 5°, and the pattern speed was differed to estimate PSE at which the two stimuli appear to have the same speed.

At the beginning of each trial, a ring for fixation (diameter 1.07°) appeared at the center and dynamically shrunk, disappearing for 0.25 s, then a fixation cross appeared at the center. In the position task, a pair of stimuli with texture motion were presented on each hemifield for 1 s (Fig. 1). The stimulus in each hemifield was horizontally positioned at one of the three eccentricities (5°, 10°, or 15°). They contained texture motion in opposite vertical directions to each other, and the directions were randomized. The vertical distance between each stimulus was varied, which was defined as the difference between stimuli with upward motion or downward motion. The vertical position difference between two stimuli was selected from 20 equally spaced values ranging from −2° to 1°. Each difference condition was repeated eight times. The order of presentation was randomized. As an exception, for two older adult participants the position difference was selected from 28 spaced values from the same range with unequal spacing (SI 1). The mean vertical position of the pair also varied randomly from −1.5° to 1.5° with 20 equally spaced steps around the fixation cross. After the disappearance of stimuli, the participants reported which stimulus was located higher.

In the speed task, participants were presented a test stimulus in a Gaussian contrast envelope and a reference stimulus in a pillbox envelope for 1 s. The test and the reference stimuli were placed randomly in each hemifield (Fig. 1). The test stimulus was presented at one of three horizontal eccentricities (5°, 10°, or 15°; visual field selected at random), whereas the reference stimulus was fixed at 5° eccentricity in the opposite visual field. The texture speed of the test stimulus was fixed as 10°/s, but that of the reference stimulus was randomly chosen from 4°/s to 13°/s with 10 equally spaced steps. Each condition was repeated 16 times. Participants reported which stimulus showed a faster motion. For both position and speed tasks, feedback was not provided to participants.

### Data analysis

We fitted cumulative Gaussian functions to measure the bias (i.e., the size of the MIPS or the size of speed bias) and variability of the responses in both the position and speed tasks. The bias was estimated by the *mu* of the Gaussian function and the variability was estimated by the *sigma* of the Gaussian function. In addition to the *mu* and *sigma* parameters, we estimated the lapse-rate parameter that represents the probability of an arbitrary response due to the lack of attention. To improve the robustness of data fitting, we applied a hierarchical Bayesian model with the assumption that the parameters for individual participants in the same age group follow a specific group-level distribution. The t-distribution, gamma distribution, and left-truncated Gaussian distribution were used for the group-level distribution for *mu*, *sigma*, and the lapse-rate parameters, respectively. The means and SDs of the t-distribution, gamma distribution, and left-truncated Gaussian distribution followed a uniform distribution and a gamma distribution, respectively. The degrees of freedom parameter of the t-distribution followed an exponential distribution. We numerically estimated the posterior distribution of each parameter by running Monte Carlo Markov Chains (MCMC), implemented in JAGS^38^ using Matlab (MathWorks, R2017a). Significance tests were conducted using RStudio (RStudio, Version 1.1). Data is available upon request.

## Results

### Position task

**Figure 2:**
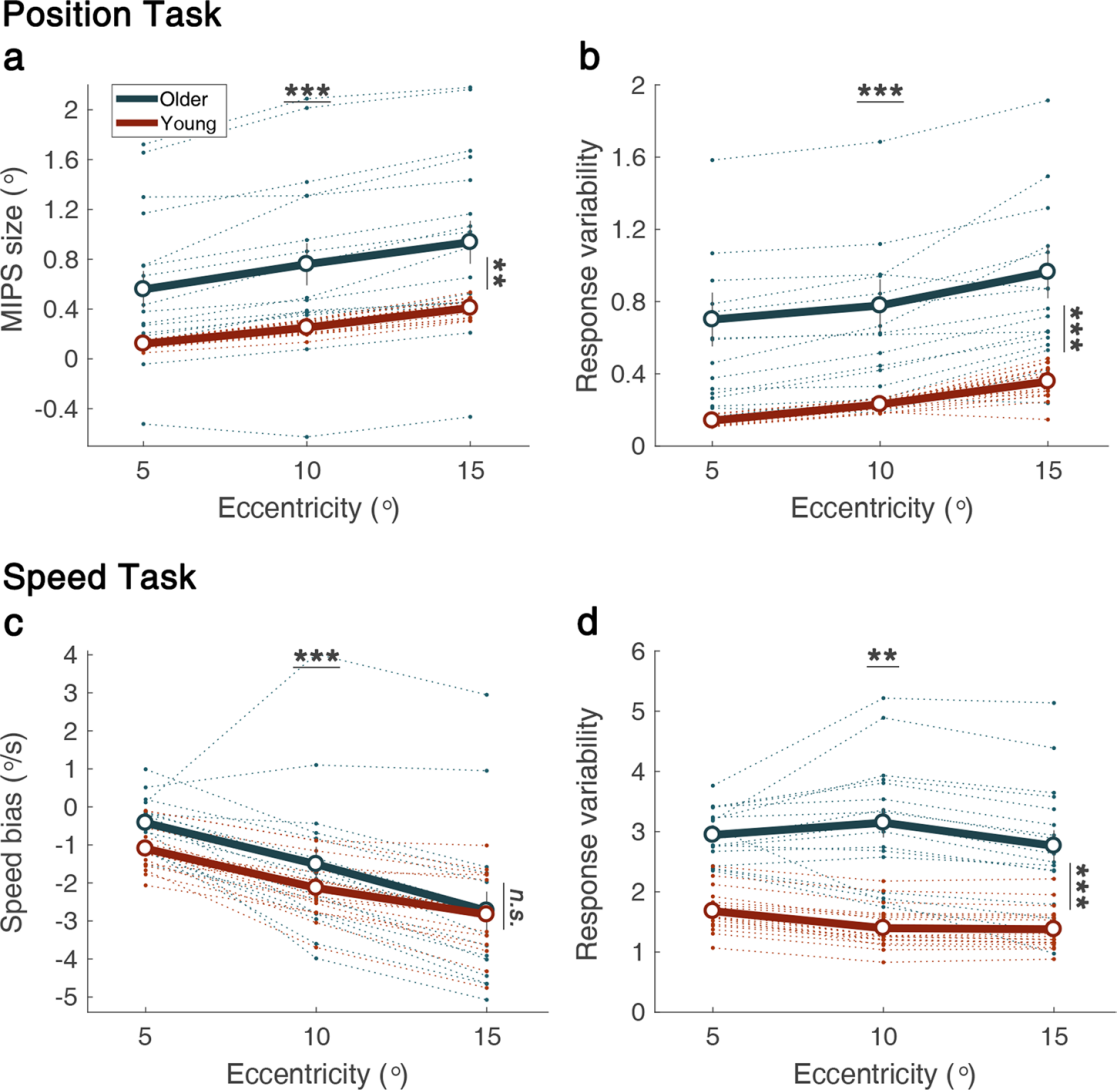
Results of Experiment 1. We measured the position shift (**a**) and response variability (**b**) of position estimation, and the speed bias (**c**) and response variability (**d**) of speed estimation from each task. (**a**) The size of the position shift by one MIPS stimulus is displayed. Here, a positive shift denotes shift to the same direction of the motion. (**b**) The noise of position estimation for one MIPS stimulus was calculated accounting for the matching ambiguity between two stimuli. (**c**) The size of the speed bias denotes the speed difference between perceived speed and physical speed. Here, negative speed bias indicates slow speed bias. (**d**) This shows the noise of speed estimation. Error bars indicate ±1 standard error. The dotted lines represent individual data. (**p < 0.01, ***p < 0.001).

We applied a split-plot ANOVA and examined credible intervals (CI) of the posterior of group-level parameters to test the effects of aging and retinal eccentricity on the size of the MIPS (Fig. 2a). As the eccentricity of stimulus increased from 5° to 15°, overall mean of the MIPS size increased from 0.34° to 0.67° and the increase was statistically significant, *F*_2*,68*_ = 158.53, *p* < 0.001, *η*_*p*_^2^ = 0.83. Consistent with the results of ANOVA, the 95% credible intervals (CI) for the MIPS size difference between 5° eccentricity and 15° eccentricity did not include zero in both young [0.24, 0.33] and older [0.24, 0.51] groups indicating significant increase of the MIPS size. These results are consistent with the findings of previous studies^31–34^.

The results of ANOVA showed that the effect of aging on the size of the MIPS was statistically significant, *F*_*1,34*_ = 10.24, *p* = 0.0030, *η*_*p*_^2^ = 0.23. In order to examine the effect of ageing without equal variance assumption across groups, we additionally applied Welch’s t-test. Being consistent with the result of ANOVA, the effect of ageing was statistically significant, *t*_*16.15*_ = 3.02, *p* = 0.008, *d* = 1.07. On average, the size of the MIPS in older adults was 2.87 times larger than young adults (mean of older adults = 0.75°, mean of young adults = 0.26°). The 95% CIs for the difference in the MIPS sizes between older and young adults did not include zero in all three eccentricities (5°: [0.09, 0.75], 10°: [0.15, 0.84], 15°: [0.15, 0.87]), which is consistent with the results of ANOVA.

The interaction effect between age and eccentricity was statistically significant, *F*_2*,68*_ = 3.331, *p* = 0.042, *η*_*p*_^2^ = 0.09, which may reflect that the increase of the MIPS size over eccentricity was relatively larger in older adults. Slope of the regression line for the MIPS size on eccentricity was steeper in older adults than in young adults, which was marginally significant, Welch’s t-test: *t*_*17.78*_ = 1.78, *p* = 0.092, *d* = 0.63. However, we could not find a corresponding interaction effect in the hierarchical Bayesian analysis. The 95% CIs for the age group difference in the MIPS size differences between eccentricities 5° and 15° included zero [−0.05, 0.23].

We then analyzed the response variability (Fig. 2b). Results of split-plot ANOVA showed that the overall mean of response variability increased as eccentricity increased and the effect was statistically significant, *F*_2*,68*_ = 80.54, *p* < 0.001, *η*_*p*_^2^ = 0.71. Being consistent with the result of ANOVA, the 95% CIs for the *sigma* difference between eccentricities 5° and 15° did not include zero in the young [0.11, 0.21] and the older [0.07, 0.31] groups. The response variability was larger in the older adult group and the difference was statistically significant (Fig. 2b), *F*_*1,34*_ = 17.29, *p* = 0.0021, *η*_*p*_^2^ = 0.34. Welch’s t-test also showed that there was a statistically significant difference between mean *sigma* across three eccentricities between the two age groups, *t*_*16.13*_ = 3.93, *p* = 0.0012, *d* = 1.39. The 95% CIs for the difference in *sigma* between older and young adults did not include zero in all three eccentricities (5°: [0.35, 1.06], 10°: [0.32, 1.05], 15°: [0.34, 1.14]). The interaction between age and eccentricity was not statistically significant, *F*_2*,68*_ = 1.31, *p* = 0.28, *η*_*p*_^2^ = 0.04. No significant effect of interaction was identified by the examination of CIs.

### Speed task

We applied a split-plot ANOVA and examined CIs of the posterior of group-level parameters to test the effects of aging and retinal eccentricity on perceived speed bias, which denotes the perceived speed of the target stimulus relative to the perceived speed of the reference stimulus (Fig. 2c). As the eccentricity of target stimulus increased from 5° to 15°, the mean perceived speed bias increased from −0.76°/s to −2.78°/s, *F*_*2,68*_ = 72.45, *p* < 0.001, *η*_*p*_^*2*^ = 0.68. Consistent with the results of ANOVA, the 95% CIs for speed bias difference between eccentricities 5° and 15° did not include zero in both young [−2.09, −1.37] and older [−3.32, −1.68] groups. This replicated the findings of previous studies showing the decrease in perceived speed in peripheral vision^34–36^. The effect of aging was not statistically significant, *F*_*1,34*_ = 1.84, *p* = 0.17, *η*_*p*_^*2*^ = 0.04. Welch’s t-test also showed that the effect of aging was not statistically significant, *t*_*22.27*_ = 1.20, *p* = 0.24, *d* = 0.41. Consistent with the results of Welch’s t-test, the 95% CIs for the difference in mean speed biases across three eccentricities between older and young adults included zero [−0.38, 1.07]. Note that this result does not necessarily indicate the absence of an aging effect on perceived speed, because the perceived speed bias reported here is relative to the perceived speed of the reference stimulus. The interaction between age and eccentricity was not statistically significant, *F*_*2,68*_ = 1.84, *p* = 0.17, *η*_*p*_^*2*^ = 0.05. No significant effect of interaction was identified by the examination of CIs, although the CIs for the difference in speed biases between older and young adults did not include zero in eccentricity 5° and did include zero in other two eccentricities (5°: [0.15, 1.22], 10°: [−0.47, 1.38], 15°: [−1.09, 0.90]).

We then analyzed the response variability (Fig. 2d). There was a statistically significant main effect of eccentricity on the response variability, *F*_*2,68*_ = 6.50, *p* = 0.0026, *η*_*p*_^*2*^ = 0.16, which reflects that the response variability generally decreased as eccentricity increased. Results of linear regression applied to individual participants’ data showed that the mean slope representing the relation between eccentricity and the response variability was below zero and the difference was statistically significant, *t*_35_ = −2.71, *p* = 0.010, *d* = −0.45. The response variability was significantly larger in the older adult group (Fig. 2d)), *F*_*1,34*_ = 52.25, *p* < 0.001, *η*_*p*_^2^ = 0.61. Welch’s t-test also showed that there was a statistically significant difference between overall mean of response variability across eccentricities between the two age groups, *t*_2*0.8*2_ = 6.94, *p* < 0.001, *d* = 2.41. Consistently, the 95% CIs for the difference between older and young adults did not include zero in all three eccentricities (5°: [0.83, 1.86], 10°: [1.11, 2.51], 15°: [0.68, 2.18]). The interaction between age and eccentricity was also statistically significant, *F*_2*,68*_ = 6.25, *p* = 0.0032, *η*_*p*_^2^ = 0.16. However, we could not find a corresponding interaction effect in the analysis of CIs [−0.54, 0.75]. Results of Turkey’s HSD test applied to each group’s data revealed that the response variability showed a statistically significant difference (*p* < 0.05) between 5° and 10°, and between 5° and 15° eccentricities in the young adults’ group, while the response variability showed a statistically significant difference between 10° and 15° eccentricities in older adults’ group. Analysis of CIs show that the 95% CIs for the difference between eccentricities 5° and 15° did not include zero in the young group [−0.49, −0.10], but it included in the older group [−0.81, 0.43].

### Correlation between the MIPS and slow speed bias

The MIPS increased and the perceived speed bias increased negatively as eccentricity increased in both young and older adults (Fig. 2a,c). These results are consistent with the prediction of the optimal tracking model that suggests the MIPS and slow speed bias are negatively related. We further tested the prediction of the model, exploring individual differences. In other words, we examined whether the MIPS size of individuals is correlated with the speed bias of individuals. As mentioned above, the measured speed bias is not the absolute speed bias, but the measured perceived speed relative to the perceived speed of the reference stimulus. Given that the perceived speed of the reference stimulus is not known, it is not meaningful to examine the relation between the MIPS and slow speed bias directly. For example, it is possible that the measured speed bias in Fig. 2c is relatively lower in young adults than older adults. It is because the perceived speed of the reference stimulus could be lower in older adults^39^. Therefore, we compared the changes in the MIPS size and the speed bias between eccentricities to cancel the effect of the perceived speed of the reference stimulus.

**Figure 3:**
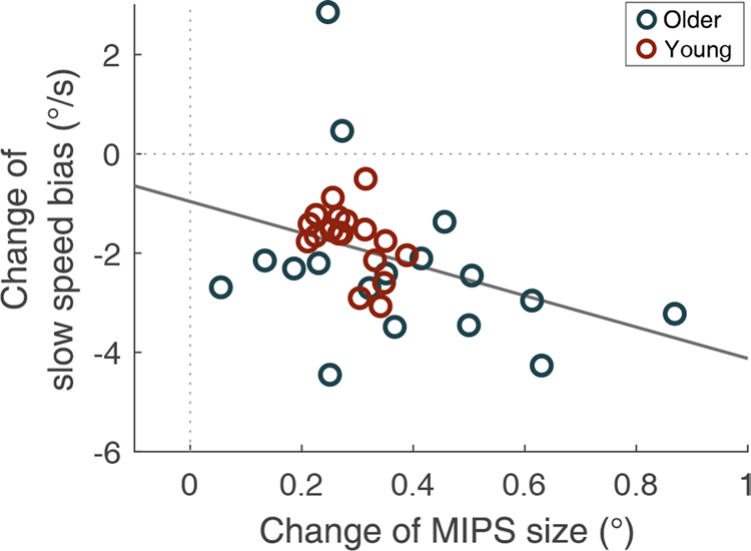
Changes of the MIPS and slow speed bias as eccentricity increases from 5**°** to 15**°**. Each point represents an individual. The MIPS increased in all individuals and the perceived speed decreased in all but two individuals as results of eccentricity change. The negative correlation between the changes of the MIPS and slow speed bias was statistically significant (*p* = 0.027). The grey solid line is the best fitting linear regression line.

Figure 3 shows the changes in the MIPS and the speed bias between two eccentricities (5° and 15°). The MIPS increased in all participants and perceived the speed bias decreased in all but two participants as eccentricity increased, which shows the general trends of negative association between the MIPS and the speed bias. At the individual level, we found a negative correlation between the changes in the MIPS and the speed bias, which was statistically significant, *r* = −0.37, *p* = 0.027. Participants who showed a larger increase in the MIPS showed a larger decrease in the speed bias as a function of eccentricity.

## Discussion

We found that the older adult participants showed the notably larger MIPS than younger participants. The MIPS demonstrates the influence of motion signal on position estimation, and the increased MIPS size in older adults indicated a stronger influence of motion signals on position estimation. This result allows us to rule out the possibilities that ageing would severely reduce the interaction between motion and position signals or that ageing would reduce the sensitivity to motion signals more severely than the sensitivity to position signals. Our finding suggests that older adult participants depended more on predictive motion signals when estimating object positions. The stronger dependency on motion signals may be because motion signals are relatively more reliable than position signals in older adults. Indeed, our data showed that the response variability of the older adult participants in the position task was 3.5 times larger than that of the younger participants, whereas the response variability of the older adult participants in the speed task was 2 times larger than that of the younger participants. In order to statistically examine the different effects of aging on the position and speed tasks, we applied a split-plot ANOVA to z-scores of position and speed data. Results showed that there was a statistically significant interaction effect between age and task factors, *F*_*1,34*_ = 5.12, *p* = 0.03, *η*_*p*_^*2*^ = 0.10, which implied that the response variability increased more in position task than in speed task by ageing.

It should be noted that the response variability cannot be a direct measure of the sensory noise. For example, the response variability of position estimation can be affected by not only the sensory noise of position signal but also the sensory noise of motion signal, and the noise involved in signal integration and perceptual decision making. However, it is also reasonable to assume that response variability of position tasks reflects the noise of position signals more than the noise of motion signals and the response variability of speed task reflects the noise of motion signals more than the noise of position signals. Therefore, the more substantial increase of response variability in position task supports the claim that motion signals are relatively more reliable than position signals in older adults.

## Experiment 2

We measured the size and response variability of the MIPS as a function of stimulus duration to examine the difference in temporal dynamics between older and young adults. Specifically, it was of interest to test whether older adults integrate sensory signals for a longer duration than young adults and, as a consequence, the MIPS size and response variability approach asymptote at a longer stimulus duration. The results do not show any significant difference in temporal patterns between the two groups. We further analyzed the changes in response variability to estimate sensory noise and internal propagation noise separately. The results suggest that sensory noise is the main factor responsible for the difference in response variability of the MIPS.

## Methods

### Participants

10 older adults (mean age: 69.9 ± 5.3 years, range: 61–76 years; 2 male) and 8 young adults (mean age: 21.9 ± 1.0 years, range: 21–23 years; 5 male) participated in the experiment. All of the older adult participants in Experiment 2 were recruited from those who participated in Experiment 1. All young participants were naïve. According to a power analysis, the minimum effect size that could have been reliably detected (*alpha* = 0.05, *power* = 0.8) with the current sample size was 1.42. Given standard deviations of the observed data, the minimum effect sizes are corresponding to 31.10 ms of mean difference between groups for ***t***_***1***_ and 123.73 ms for ***t***_***2***_

### Stimuli and apparatus

The experimental settings were identical to those for the position task in Experiment 1, except for the following. The stimulus duration varied from 31 ms to 1000 ms at seven discrete levels (31, 55, 98, 175, 313, 559, 1000 ms), and the eccentricity of stimulus was fixed at 10°. We added a control condition in which a stimulus with a stationary texture was presented for 31 ms. The projector refresh rate was 360 Hz. A total of 800 trials were conducted in five blocks.

### Data analysis

As in Experiment 1, we fitted cumulative Gaussian functions to measure the bias and variability of participants’ responses in eight stimulus duration conditions (SI 3). To improve the robustness of data fitting, we applied a hierarchical Bayesian model.

## Results

**Figure 4:**
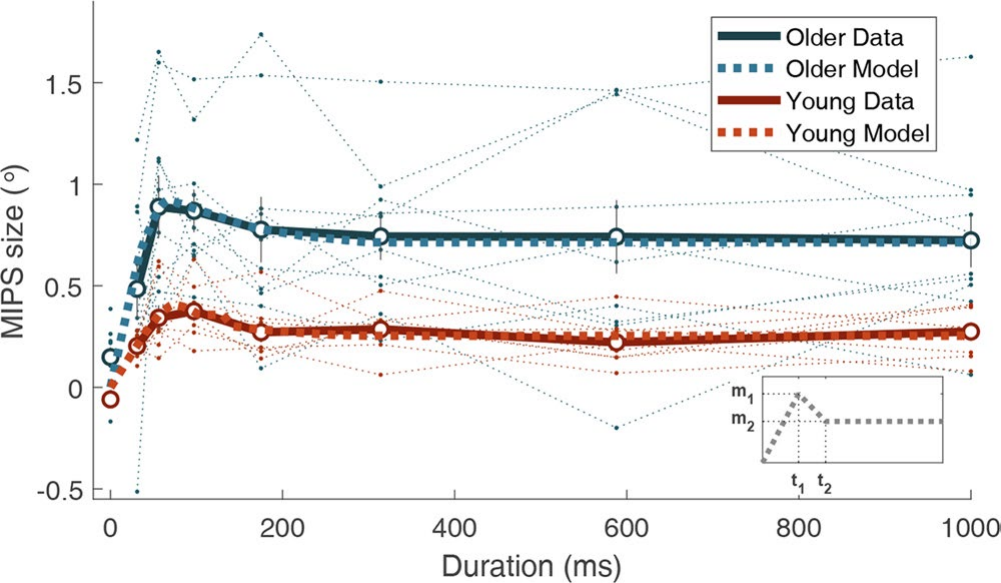
Temporal evolution of position shift. Each dot indicates group mean for shift, and error bars indicate ±1 standard error. The polygonal line was fitted individually, and the averaged individual fitted lines are presented as thick dotted lines. The thin dotted lines show individual shifts. The data at 0 ms correspond to the control condition with static texture presented for 31 ms.

Young and older adults showed similar patterns of the MIPS changes as a function of stimulus duration (Fig. 4). In both groups, the MIPS initially peaked at around 56–97 ms and decreased to converge at a lower level. To quantify the temporal changes of the MIPS size, we fit three lines to individuals’ data. Three lines connected the origin (0, 0), the first bending point (***t***_***1***_, ***m***_***1***_), the second bending point (***t***_***2***_, ***m***_***2***_), and the level point (1000, ***m***_***2***_). Four parameters (***t***_***1***_, ***m***_***1***_, ***t***_***2***_, ***m***_***2***_) were estimated for each individual (Fig. 4). The means of estimated ***m***_***1***_ and ***m***_***2***_ were 0.46° and 0.25° in young adults and 1.00° and 0.71° in older adults. The means of estimated ***t***_***1***_ and ***t***_***2***_ were 76 ms and 180 ms in young adults and 56 ms and 183 ms in older adults.

We applied Welch’s t-test to examine group differences. Among the four parameters, the two parameters representing the size of the MIPS showed statistically significant differences between groups (***m***_***1***_: *t*_*13.48*_ = 3.65, *p* = 0.0028, *d* = 1.61; ***m***_***2***_: *t*_*9.88*_ = 3.45, *p* = 0.0063, *d* = 1.47), whereas the two parameters representing temporal points did not (***t***_***1***_: *t*_*10.9*7_ = −1.77, *p* = 0.10, *d* = −0.87; ***t***_***2***_: *t*_*16.00*_ = 0.083, *p* = 0.94, *d* = 0.038). We then applied a split-plot ANOVA with age as a between group factor and the MIPS size at temporal change points (***m***_***1***_ and ***m***_***2***_) as a within group factor to further characterize the change between ***m***_***1***_ and ***m***_***2***_. There were statistically significant differences between age groups, *F*_*1,16*_ = 11.03, *p* = 0.0043, *η*_*p*_^*2*^ = 0.41, and between ***m***_***1***_ and ***m***_***2***_, *F*_*1,16*_ = 65.48, *p* < 0.001, *η*_*p*_^*2*^ = 0.80. The decreases from ***m***_***1***_ to ***m***_***2***_ were statistically significant in both young, *t*_*7*_ = 4.38, *p* = 0.0033, *d* = 1.51, and older adults, *t*_9_ = 7.07, *p* < 0.001, *d* = 0.70. The interaction between the age factor and temporal change points was not statistically significant, *F*_*1,16*_ = 0.95, *p* = 0.34, *η*_*p*_^2^ = 0.06. Overall, older adults showed the significantly larger MIPS size than young adults as in Experiment 1, however the patterns of temporal changes were similar between groups.

We then examined the initial rate of the increase of the MIPS size. The rates of the increase estimated at 31 ms and 55 ms were averaged to improve the reliability of estimation, given that, as one can visually verify, the rates of the increase were comparable at 31 ms and 55 ms conditions (young: 6.57 and 6.12 °/s, old: 15.64 and 15.87 °/s). Note that we did not use ***m***_***1***_/***t***_***1***_ as a measure of the initial rate of the increase, because a division by a near-zero noisy estimate (***t***_***1***_) causes a large variability. Older adults showed a higher rate of the increase than young adults, which was statistically significant, Welch’s t-test: *t*_*10.00*_ = 3.00, *p* = 0.013, *d* = 1.27. This result suggests that a stronger influence of motion was exerted on position estimation for older adults. An unexpected result was that the rate of increase in older adults (15.75 °/s) was higher than the physical texture speed (10 °/s). Although the difference was not statistically significant, *t*_9_ = 1.88, *p* = 0.09, *d* = 0.60, we could not exclude the possibility that the participants reconstructed the percept of stimulus position after the disappearance of stimulus by extrapolating the position along the texture motion direction for longer than the stimulus duration^40^. The rate of increase in young adults (6.34 °/s) was lower than the physical texture speed (10 °/s), which was statistically significant, *t*_7_ = −5.05, *p* = 0.0015, *d* = −1.79. Unlike older participants, young participants did not attribute the texture motion entirely to the object motion.

**Figure 5:**
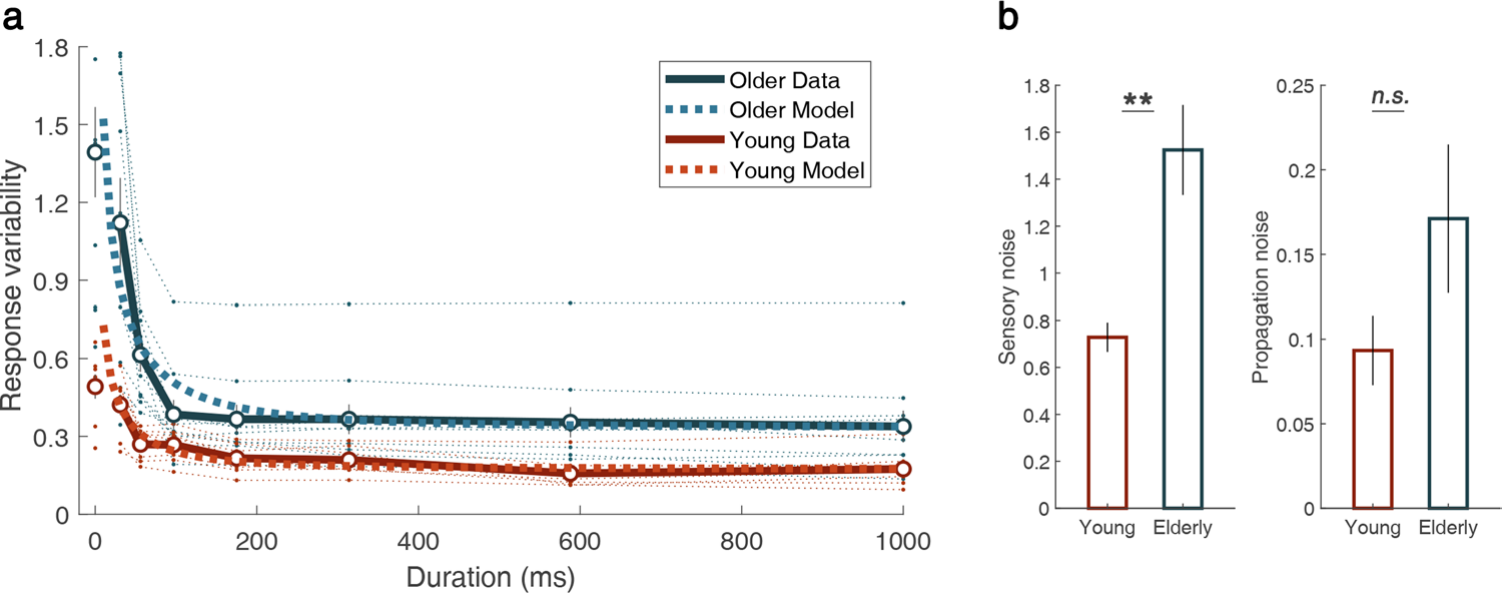
(**a**) Temporal evolution of position estimation noise. The open dots indicate the group mean for estimated noise; error bars indicate ±1 standard error. The optimal integration model was fitted individually, and averaged lines are shown as thick dotted lines. The thin dotted lines show individual noises. The data at 0 ms correspond to the control condition with static texture presented for 31 ms. (**b**) Parameters of the model for individual data. Group means for sensory and propagation noise are presented. (**p < 0.01).

The response variability decreased as the stimulus duration increased in both groups (Fig. 5a). We quantified the decrease by fitting an exponential function to individuals’ data:

1 $$\,{{\boldsymbol{\sigma }}}_{{\boldsymbol{t}}}={\boldsymbol{a}}\ast {e}^{-{\boldsymbol{bt}}}+{\boldsymbol{c}}$$

where (***a*** **+** ***c)*** represents the initial value, ***b*** represents the decay ratio, and ***c*** represents the asymptote. Welch’s t-test revealed that the parameters ***a*** and ***c****,* which reflect the size of the response variability*,* were significantly larger in older adults than in young adults (***a***: *t*_*15.85*_ = 2.25, *p* = 0.039, *d* = 1.05; ***c***: *t*_*14.83*_ = 3.81, *p* = 0.0017, *d* = 1.71). However, the effect of age was not statistically significant for the parameter ***b*** (***b***: *t*_*15.37*_ = 0.90, *p* = 0.38, *d* = 0.43), which determines the shape of decaying curve. Older adults showed significantly larger response variability than young adults, however the patterns of temporal changes were similar between groups.

Decreasing response variability implies that sensory signals are integrated over time. In order to characterize the changes in response variability, we further examined the sources of the response variability. In addition to the sensory noise, temporal integration involves unavoidable internal propagation noise. The internal propagation noise represents the increase of estimation variability between time steps due to, for example, memory noise or prediction error. If the internal propagation noise is relatively small compared to the sensory noise, an optimal integrator would integrate the sensory signals for a longer duration and vice versa^41^. Assuming that the visual system optimally integrates sensory signals over time, we fitted an optimal integrator model with sensory noise and propagation noise as free parameters. Sensory noise and internal propagation noise are assumed to follow Gaussian distribution with zero mean and variances ${{\boldsymbol{\sigma }}}_{{\boldsymbol{s}}}^{2}$ and ${{\boldsymbol{\sigma }}}_{{\boldsymbol{p}}}^{2}$, respectively (Eq. 2).

Suppose that variance of the position percept at time ***t*** is ${{\boldsymbol{\sigma }}}_{{{\boldsymbol{n}}}_{{\boldsymbol{t}}}}^{2},$ and the variance of responses measured through the task is ${{\boldsymbol{\sigma }}}_{{{\boldsymbol{m}}}_{{\boldsymbol{t}}}}^{2}$. The variance of estimation at the next time step ***t*** + ***1*** can be formalized as

2 $${{\boldsymbol{\sigma }}}_{{{\boldsymbol{n}}}_{{\boldsymbol{t}}+1}}^{2}={\left(\frac{{{\boldsymbol{\sigma }}}_{{\boldsymbol{s}}}^{2}}{{{\boldsymbol{\sigma }}}_{{{\boldsymbol{n}}}_{{\boldsymbol{t}}}}^{2}+{{\boldsymbol{\sigma }}}_{{\boldsymbol{p}}}^{2}+{{\boldsymbol{\sigma }}}_{{\boldsymbol{s}}}^{2}}\right)}^{2}({{\boldsymbol{\sigma }}}_{{{\boldsymbol{n}}}_{{\boldsymbol{t}}}}^{2}+{{\boldsymbol{\sigma }}}_{{\boldsymbol{p}}}^{2})+{\left(\frac{{{\boldsymbol{\sigma }}}_{{{\boldsymbol{n}}}_{{\boldsymbol{t}}}}^{2}+{{\boldsymbol{\sigma }}}_{{\boldsymbol{p}}}^{2}}{{{\boldsymbol{\sigma }}}_{{{\boldsymbol{n}}}_{{\boldsymbol{t}}}}^{2}+{{\boldsymbol{\sigma }}}_{{\boldsymbol{p}}}^{2}+{{\boldsymbol{\sigma }}}_{{\boldsymbol{s}}}^{2}}\right)}^{2}\cdot {{\boldsymbol{\sigma }}}_{{\boldsymbol{s}}}^{2}$$

3 $${{\boldsymbol{\sigma }}}_{{{\boldsymbol{m}}}_{{\boldsymbol{t}}+1}}^{2}={\left(\frac{{{\boldsymbol{\sigma }}}_{{\boldsymbol{s}}}^{2}}{{{\boldsymbol{\sigma }}}_{{{\boldsymbol{n}}}_{{\boldsymbol{t}}}}^{2}+{{\boldsymbol{\sigma }}}_{{\boldsymbol{p}}}^{2}+{{\boldsymbol{\sigma }}}_{{\boldsymbol{s}}}^{2}}\right)}^{2}\cdot {{\boldsymbol{\sigma }}}_{{{\boldsymbol{m}}}_{{\boldsymbol{t}}}}^{2}+{\left(\frac{{{\boldsymbol{\sigma }}}_{{{\boldsymbol{n}}}_{{\boldsymbol{t}}}}^{2}+{{\boldsymbol{\sigma }}}_{{\boldsymbol{p}}}^{2}}{{{\boldsymbol{\sigma }}}_{{{\boldsymbol{n}}}_{{\boldsymbol{t}}}}^{2}+{{\boldsymbol{\sigma }}}_{{\boldsymbol{p}}}^{2}+{{\boldsymbol{\sigma }}}_{{\boldsymbol{s}}}^{2}}\right)}^{2}\cdot {{\boldsymbol{\sigma }}}_{{\boldsymbol{s}}}^{2}$$

assuming that the system optimally integrates the signals given the internal propagation noise^41^. The variance of responses is not affected by the uncertainty of prior which is propagated estimation from the previous time step. By iteratively applying the variance update function, we simulated temporal changes in response variability and estimated ${{\boldsymbol{\sigma }}}_{{\boldsymbol{s}}}^{2}$, and ${{\boldsymbol{\sigma }}}_{{\boldsymbol{p}}}^{2}$ for individual participants. We applied Welch’s t-test to examine group differences. As shown in Fig. 5b, we found that that the sensory noise of older adults was larger than that of young adults and the difference was statistically significant, *t*_*10.84*_ = 3.95, *p* = 0.0023, *d* = 1.70. The difference in the propagation noise between older and young adults was not statistically significant, *t*_*1*2*.60*_ = 1.60, *p* = 0.13, *d* = 0.70. We then applied a split-plot ANOVA to z-score of the log-transformed data with age as a between-group factor and type of noise (${{\boldsymbol{\sigma }}}_{{\boldsymbol{s}}}^{2}$ and ${{\boldsymbol{\sigma }}}_{{\boldsymbol{p}}}^{2}$) as a within-group factor. There was a significant difference between age groups, *F*_*1,16*_ = 12.44, *p* = 0.0028, *η*_*p*_^2^ = 0.44, and the interaction effect showed a tendency consistent with Welch’s t-test although it did not reach a statistically significant level, *F*_*1,16*_ = 2.37, *p* = 0.14, *η*_*p*_^*2*^ = 0.13. By and large, results suggest that the difference in the sensory noise mainly caused the sizable difference in response variability between young and older adults.

The control condition, in which the texture was static, allowed us to examine two aspects of the data. First, we could confirm that the MIPS size was not significantly different from zero, as expected, when the texture motion was absent. The CIs of the MIPS size for both young [−0.18, 0.05] and older [−0.16, 0.48] groups included zero. Second, we could estimate the position uncertainty more directly, because the response variability measured in the control condition is free from the influence of the motion signals. On average, the response variability of older adults was 2.83 times larger than that of young adults, which was smaller than the ratio measured in Experiment 1, but comparable to the ratio measured in 31 ms condition (2.67 times) in Experiment 2. Comparing with the 31 ms motion condition, the response variability was larger in the control condition with a statistically significant effect, *F*_*1,16*_ = 456.5, *p* < 0.001, *η*_*p*_^2^ = 0.96. It is to be noted that the stimulus duration of the control condition was the same as that of the 31 ms condition but with the absence of motion texture. It reflects that the reliability of position estimation was improved with the existence of motion signal.

## Discussion

We examined the size and response variability of the MIPS as a function of stimulus duration and found that older and young adults showed similar patterns of temporal dynamics, although the magnitudes of the MIPS size and response variability were significantly larger in older adults. A further analysis of the response variability using an optimal integrator model revealed that sensory noise is the main factor responsible for the magnitude difference between young and older adults.

A point in the data that need to be addressed is that the response variability in the 1 s duration condition was larger than the response variability at 10° eccentricity in Experiment 1, although they are the same condition. The difference was especially apparent in older participants group. One difference between the two experiments that might have affected the response variability is the requirement for the attention shift. In Experiment 1, three different eccentricity conditions were randomly interleaved over trials, which required participants to shift attention at the onset of stimulus. On the contrary, in Experiment 2, the eccentricity was fixed at 10°. Given that aging is known to affect the ability to quickly shift attention in space^42^, the requirement for the attentional shift in Experiment 1 might have affected the older adults’ performance more severely. Another factor that might have caused the decrease of response variability in older adults in Experiment 2 is that all older participants of Experiment 2 participated in Experiment 1 first. The experience of Experiment 1 might have trained them to reduce the response variability.

The pattern of temporal changes of the MIPS size we observed is largely consistent with prior literature. Chung *et al*. (2007) reported that the MIPS size increased with stimulus duration until the initial peak appearing around 47 ms and then decreased to reach a steady-state^43^. Arnold *et al*. (2007) showed that the MIPS size monotonically increased with stimulus duration and reached asymptote around 60 ms when naïve subjects were tested^31^. The time to reach the peak MIPS (47 ms and 60 ms) is comparable to our data (57 ms 73 ms) in both studies. In a different context, Pack and Born (2001) reported that it takes about 60 ms for MT neurons to reach a steady-state response that represents global motion directions^44^. Our results and prior literature suggest that it requires at least approximately 60 ms for the visual system to integrate relevant signals and reach a steady-state estimate of a motion stimulus. The non-monotonical changes of the MIPS size observed in our data are consistent with the data reported by Chung *et al*., while Arnold *et al*. reported a monotonic increase of the MIPS size. Further research is needed to verify the conditions that determine the shape of temporal changes of the MIPS size.

## General Discussion

Tracking moving objects is an essential function of the visual system, and failure of tracking ability causes difficulties in daily activities, such as crossing a road, driving, and playing sports^45^. An important component of tracking is the integration of position signals and predictive motion signals over time. In this study, we examined the effect of aging on the integration of position and motion signals using the MIPS phenomenon.

In Experiment 1, we found that older adult participants showed the almost 3-fold larger MIPS than younger participants, which implies that the former relied more on predictive motion signals when estimating the position of an object. We hypothesize that the relatively large noise of position signals in older adults is responsible for their increased dependency on motion signals. An analogous finding has been reported in the domain of sensory motor integration. Older adults, when asked to report the intensity of sensations, tend to rely more on their sensory motor prediction of self-generated actions than on the sensory inputs from the actions. The reliance on sensory motor predictions increases with age, in proportion to the uncertainty of sensory information^46^. Strong reliance on predictive motion signals and predictive sensory motor signals could be understood to be a rational strategy to compensate for noisy sensory signals. In the shape perception, the ability to integrate orientation and position information is not deteriorated by aging, while older adults show higher response variability in fine shape discrimination tasks^11^.

In Experiment 2, we measured the size and response variability of the MIPS as a function of stimulus duration and then applied a model of sequential signal integration to estimate the size of propagation and sensory noises separately. The older adult participants showed significantly larger sensory noise compared with the younger participants. Meanwhile, the difference in size of propagation noise between age groups did not reach statistical significance. Again, the present results indicate that the difference between older adult and younger participants can be largely explained by the deteriorated sensory signals.

The increased MIPS in older adults is likely a consequence of optimal integration, in which relatively accurate predictive motion signals are given more weight compared with noisy position signals. Notably, recent studies showed that the size of the MIPS in patients with mild Alzheimer’s disease was smaller than that in an age-matched control group and the size decreased with the severity of Alzheimer symptoms^47,48^. An intriguing explanation is that the deteriorated visual memory of Alzheimer’s patients^49,50^ might weaken the influence of predictive motion signals on position perception, resulting in the relatively small MIPS size. Given that age-related deterioration of sensory signals is unavoidable, the increasing size of the MIPS in older adults can be an indication of normal aging.

The response variability in speed estimation did not significantly change across eccentricities in both young and older adult groups, whereas the speed bias significantly decreased as eccentricity increased (Fig. 2c). The Bayesian observer model of speed perception suggests that slow speed bias is a result of statistical inference, in which noisy sensory signals are integrated with prior knowledge of the speed distribution^51^. According to the model, the size of speed bias depends on the size of sensory noise and, as a consequence, its increase is expected to be accompanied by an increase in sensory noise. Meanwhile, the optimal tracking model suggests that speed bias is mainly due to an attribution problem. Motion signal generated by texture motion is partly attributed to object motion, and perceived texture motion speed tends to decrease. According to the model, speed bias is expected to increase when uncertainties of the position or speed increase^34^. We found that the increase of speed bias was accompanied by the increase of position uncertainty without a statistically significant change in speed uncertainty in the data of both young and older adults. This result is consistent with the prediction of the optimal tracking model but contradicts the prediction of the conventional Bayesian model of speed perception.

As reported in the Method sections, the participants’ gender ratios in Experiment 1 (female/male, young: 7/14, old: 18/6) and Experiment 2 (young: 3/5, old: 8/2) were not balanced between young and older groups. In both experiments, the proportion of females was higher in the older adults’ group, and the proportion of males was higher in the younger adults’ group. This imbalance might have affected the MIPS size and response variability. According to literature, female older adults show higher thresholds in motion detection tasks than male older adults^16–18,52^, while the gender difference on spatial acuity task is not statistically significant^7,53^. Taking these results together, one can expect that female older adults will show larger response variability in the speed task and smaller illusory shift in the MIPS task. It is because the weak motion signals with large uncertainty will decrease the influence of motion on the position estimation. The gender imbalance in our experiments possibly has lowered the MIPS size and increased the response variability of the speed task in older adults as well. We could find a consistent tendency in older adults’ data (the MIPS size female: 0.71°, male: 0.88°, *SD* in speed task female: 3.05, male: 2.63), although the differences were not statistically significant (*p* > 0.22). The imbalanced gender ratio possibly has affected the estimations of the MIPS size and the response variability; however, it is unlikely that the effect increased the age difference reported in our experiments.

In both position and speed tasks of Experiment 1, older adult participants showed significantly larger response variability compared with younger participants. However, the degree of difference was not the same. The response variability in position task of older participants was 3.5 times larger than that of younger participants, whereas the response variability in speed task of older participants was 2 times larger than that of younger participants. As aging takes place, the accuracy of position signals reduces more drastically compared with that of speed signals. The results show that motion signals are likely to be more reliable than position signals for older adults, which is consistent with the hypothesis that increased MIPS in older adults is a natural consequence of depending more on reliable predictive motion signals.

Although numerous studies have examined the effects of aging on visual perception, it is largely unknown how ageing affects the way our visual system integrates position and predictive motion signals. We used the MIPS, which requires the integration of position and motion signals, to quantify the effect of ageing. Our results showed that the size of the MIPS increases considerably as one grows older, whereas the temporal changes of the MIPS as a function of stimulus duration are not affected by aging. The strong influence of motion signals on position estimation shown in our results implies that older adults are likely to depend more on predictive motion signals in visual object tracking. Increasing dependence on predictive motion signals could be a rational strategy to compensate for increased positional uncertainty of sensory signals.

## Supplementary information

Supplementary information.
