# Supplementary material for: Integration of Position and Predictive Motion Signals in Aging Vision
Source: Sci Rep. 2020 May 29;10:8783. doi: 10.1038/s41598-020-65568-y (PMC7260223; doi:10.1038/s41598-020-65568-y)
Supplement: Supplementary file 1 — Supplementary information. [file 41598_2020_65568_MOESM1_ESM.pdf]

Supplementary Information

Integration of Position and Predictive Motion Signals in Aging Vision

Hyun-Jun Jeon, Yeojeong Yun, Oh-Sang Kwon*

Department of Human Factors Engineering, Ulsan National Institute of Science and Technology (UNIST), Ulsan, 44919, Republic of Korea

*Corresponding Author

Oh-Sang Kwon

Ulsan National Institute of Science and Technology (UNIST)

Department of Human Factors Engineering

Bldg. 104 Room #1001-10

50 UNIST-gil, Ulsan, 44919, Republic of Korea

+82-52-217-2735

oskwon@unist.ac.kr

SI 1: Details of method for exceptional participants in the position task, Experiment 1

Two older adult participants ran the position task with the same range of positional difference between two stimuli as other participants, but the number of steps were 28 with unequal space between steps (Fig. S2, Participant E#1, E#2). The number of trials for each step was distributed from 4 to 12 as like the figure below. The total number of trials was 160.

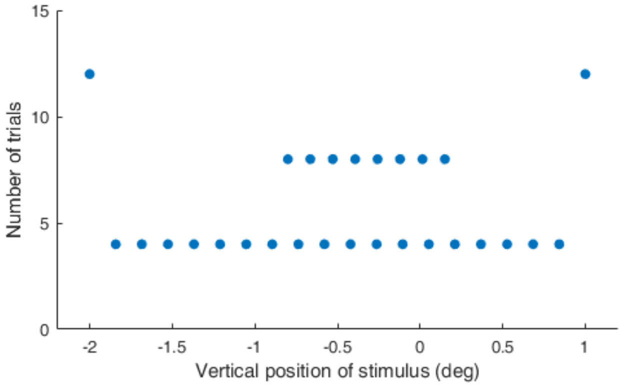

Fig. S1. Participants who ran the position task with exceptional conditions in Experiment 1.

SI 2: Response data and fitted psychometric functions in Experiment 1

- *Older adult group*

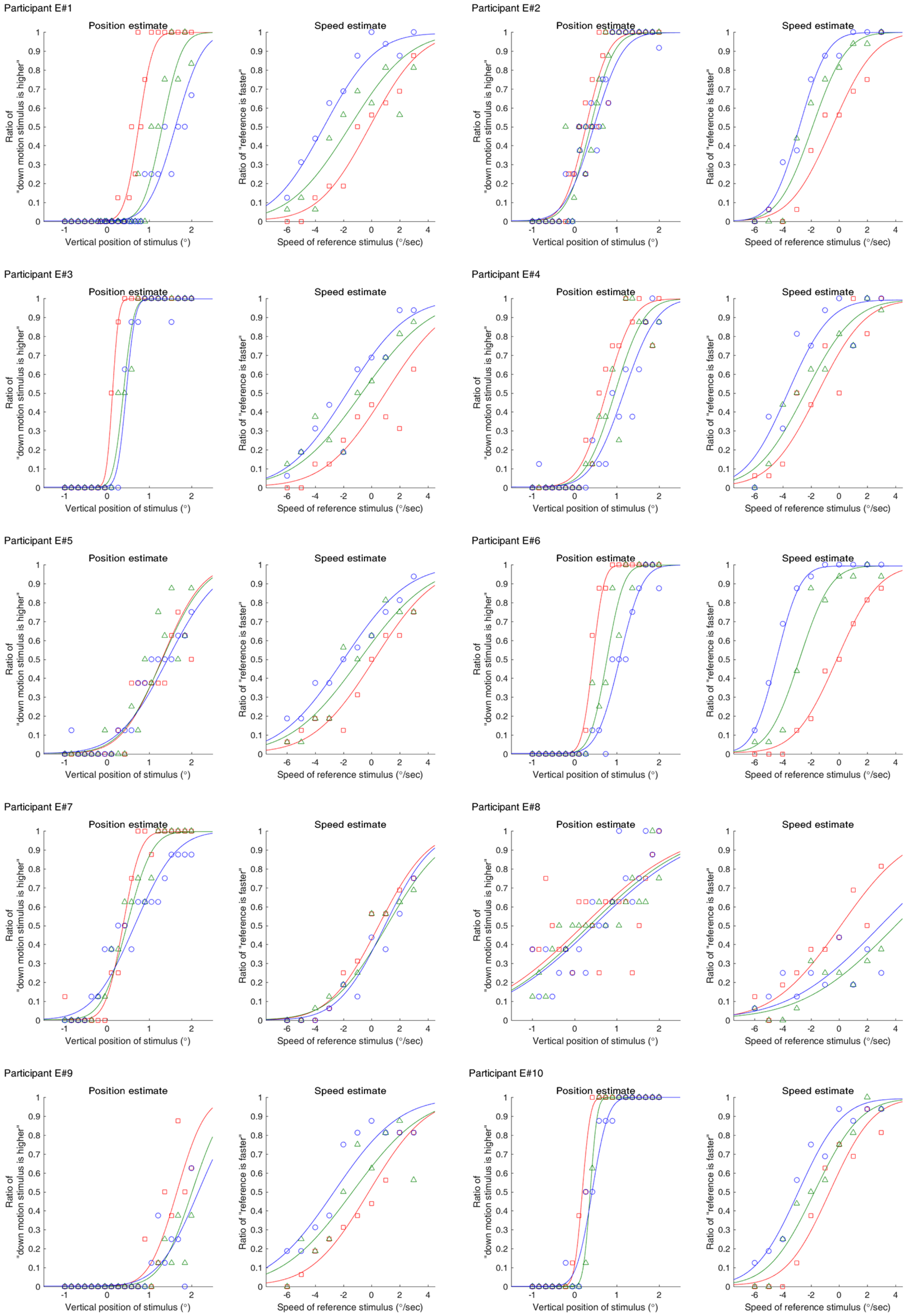

Fig. S2 (Related to Fig. 2). Response data and fitted psychometric functions of older adult participants in Experiment 1. Dots indicate the response probability to “downward motion stimulus is higher” in the position task and “reference stimulus is faster” in the speed task. Lines indicate fitted psychometric functions. Red, green, and blue colors represent eccentricities of 5°, 10°, and 15° conditions, respectively.

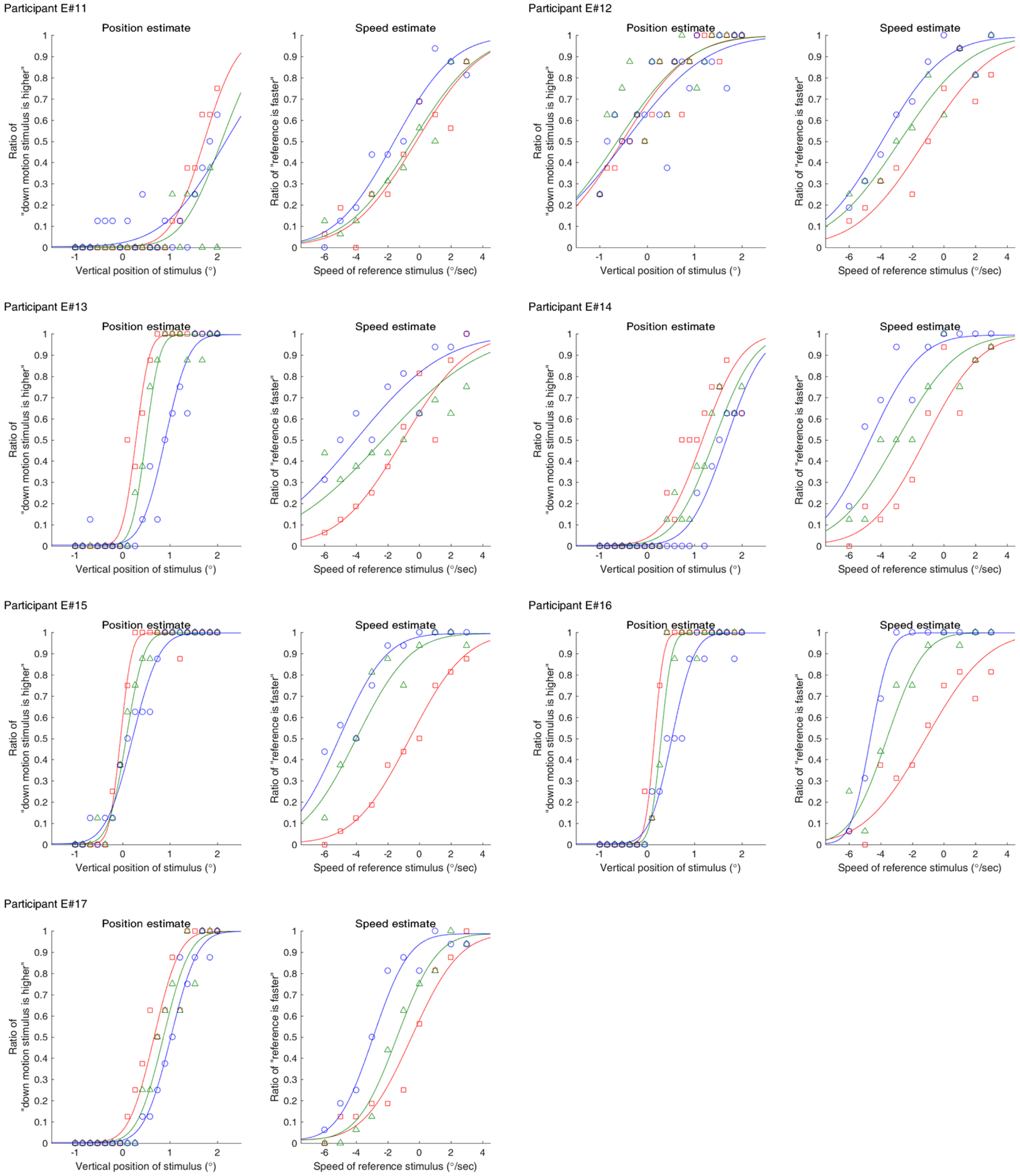

Fig. S2 (continued)

- ***Young adult group***

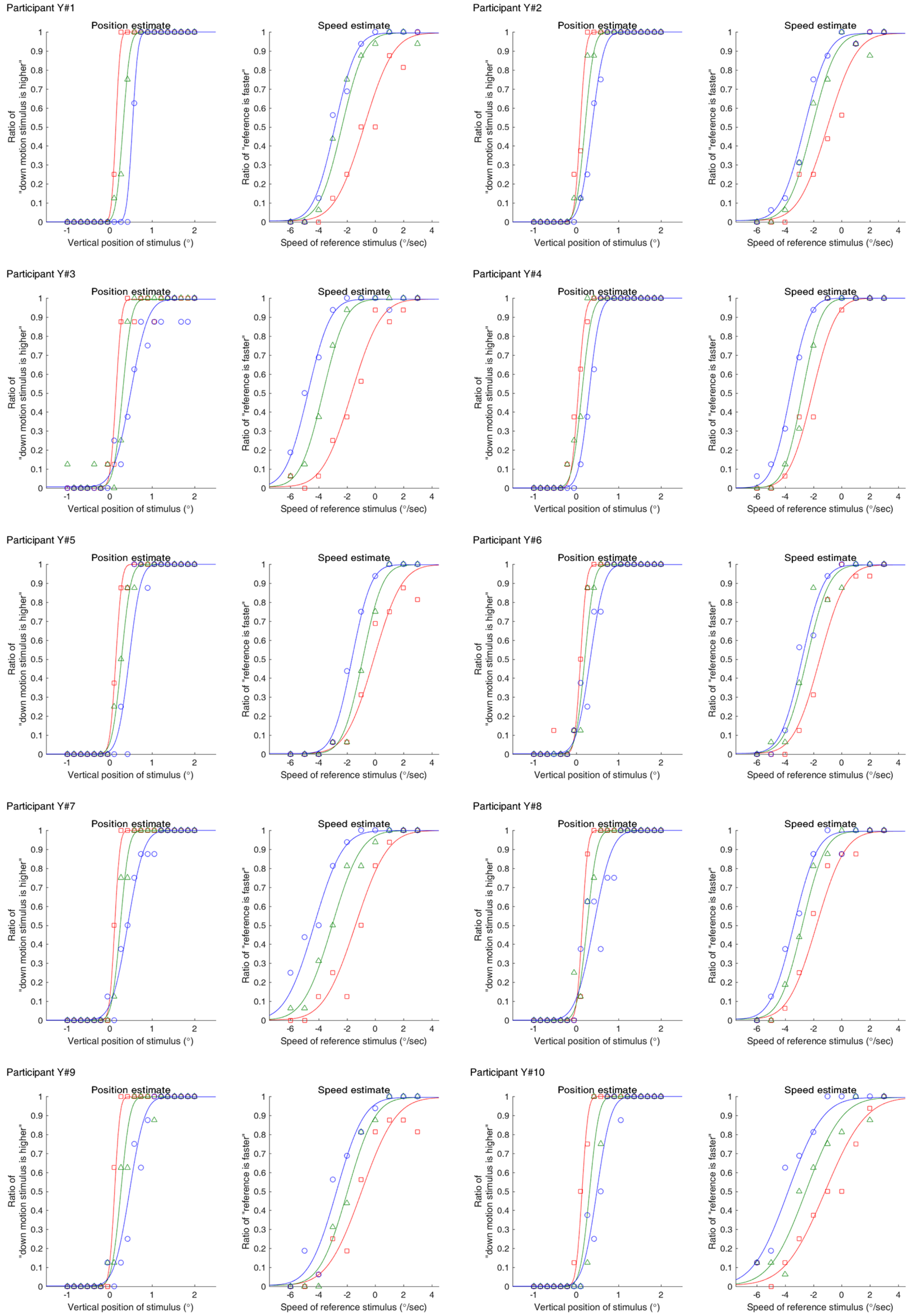

Fig. S3 (Related to Fig. 2). Response data and fitted psychometric functions of young adult participants in Experiment 1. Dots indicate the response probability to “downward motion stimulus is higher” in the position task and “reference stimulus is faster” in the speed task. Lines indicate fitted psychometric functions. Red, green, and blue colors represent eccentricities of 5°, 10°, and 15° conditions, respectively.

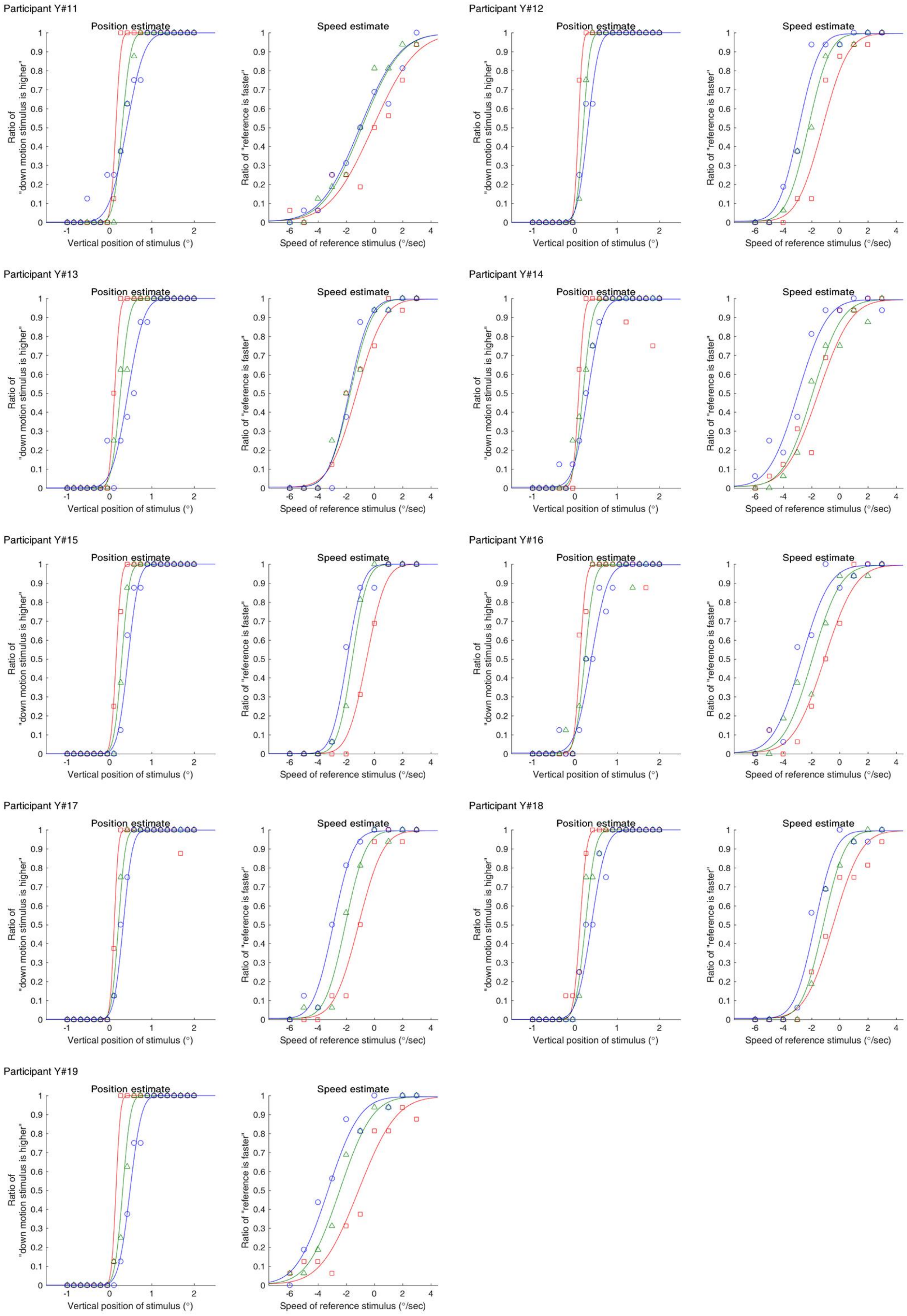

Fig. S3 (continued)

SI 3: Response data and fitted psychometric functions in Experiment 2

- *Older adult group*

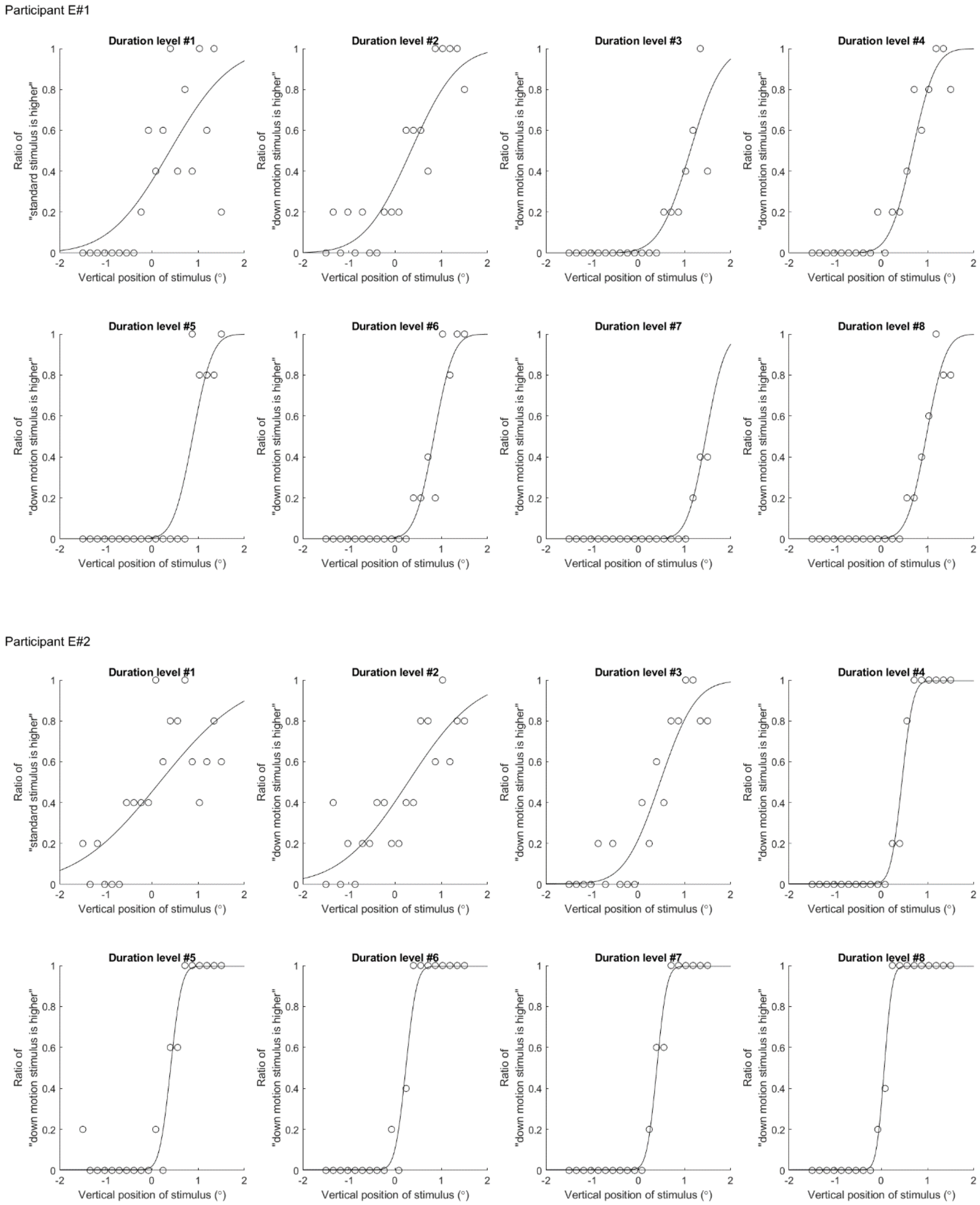

Fig. S4 (Related to Fig. 4 and Fig. 5A). Response data and fitted psychometric functions of older adult participants in Experiment 2. Dots indicate the response probability to "standard stimulus is higher" in the stationary-stimulus condition and "downward motion stimulus is higher" in the motion-stimulus condition.

The standard stimulus in the stationary-stimulus condition is vertically placed as much as the vertical position of stimulus. Lines indicate fitted psychometric functions.

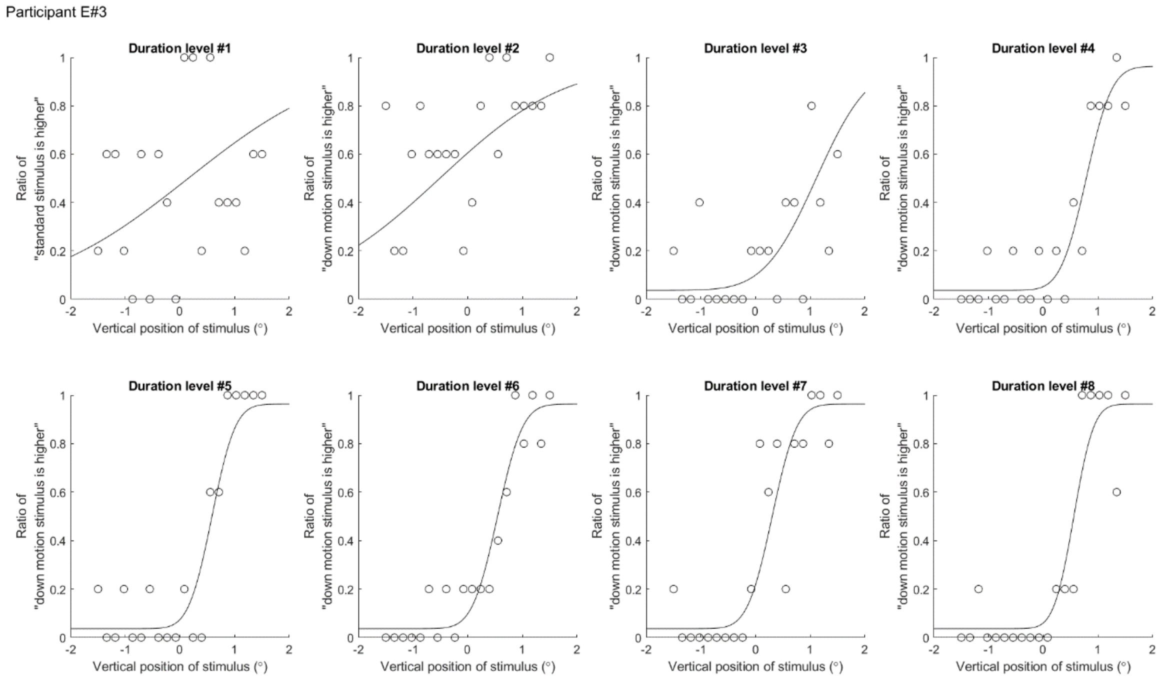

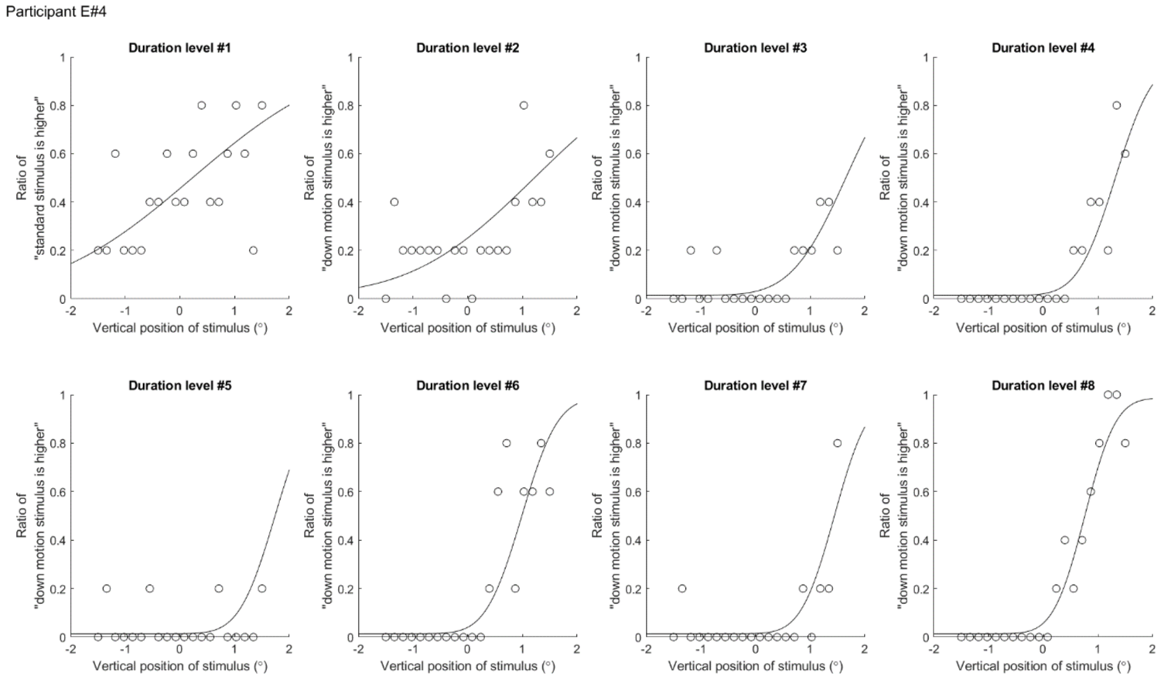

Fig. S4 (continued)

Participant E#5

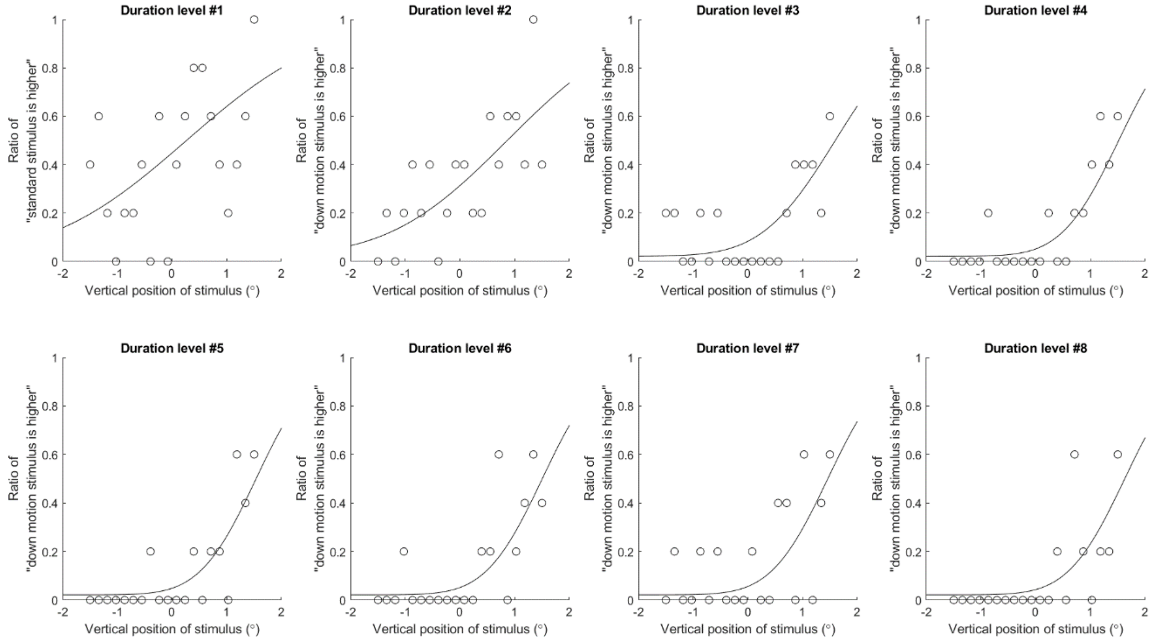

Participant E#6

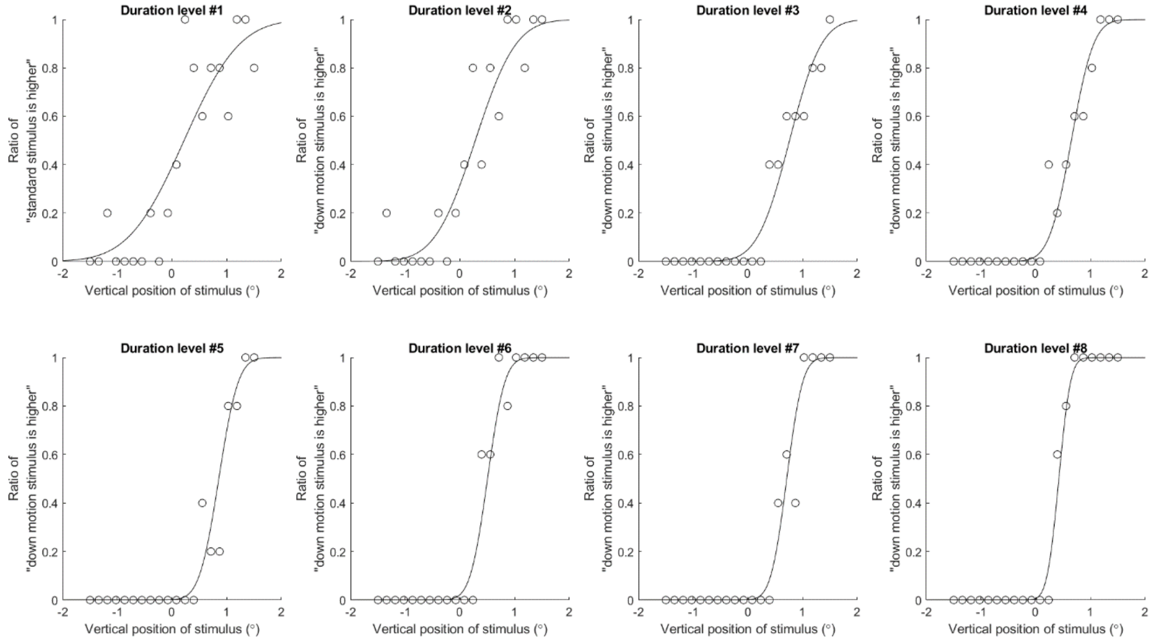

Fig. S4 (continued)

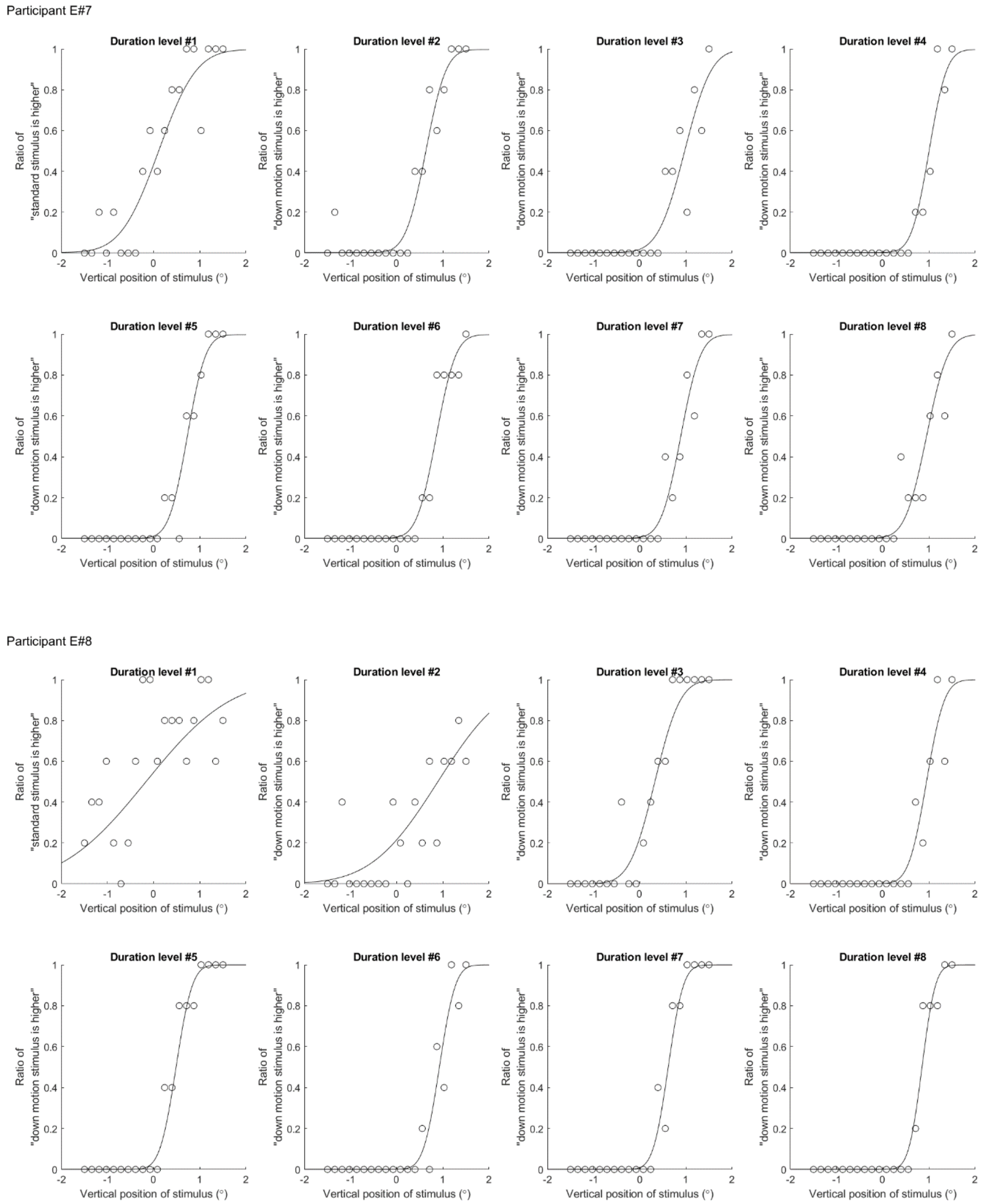

Fig. S4 (continued)

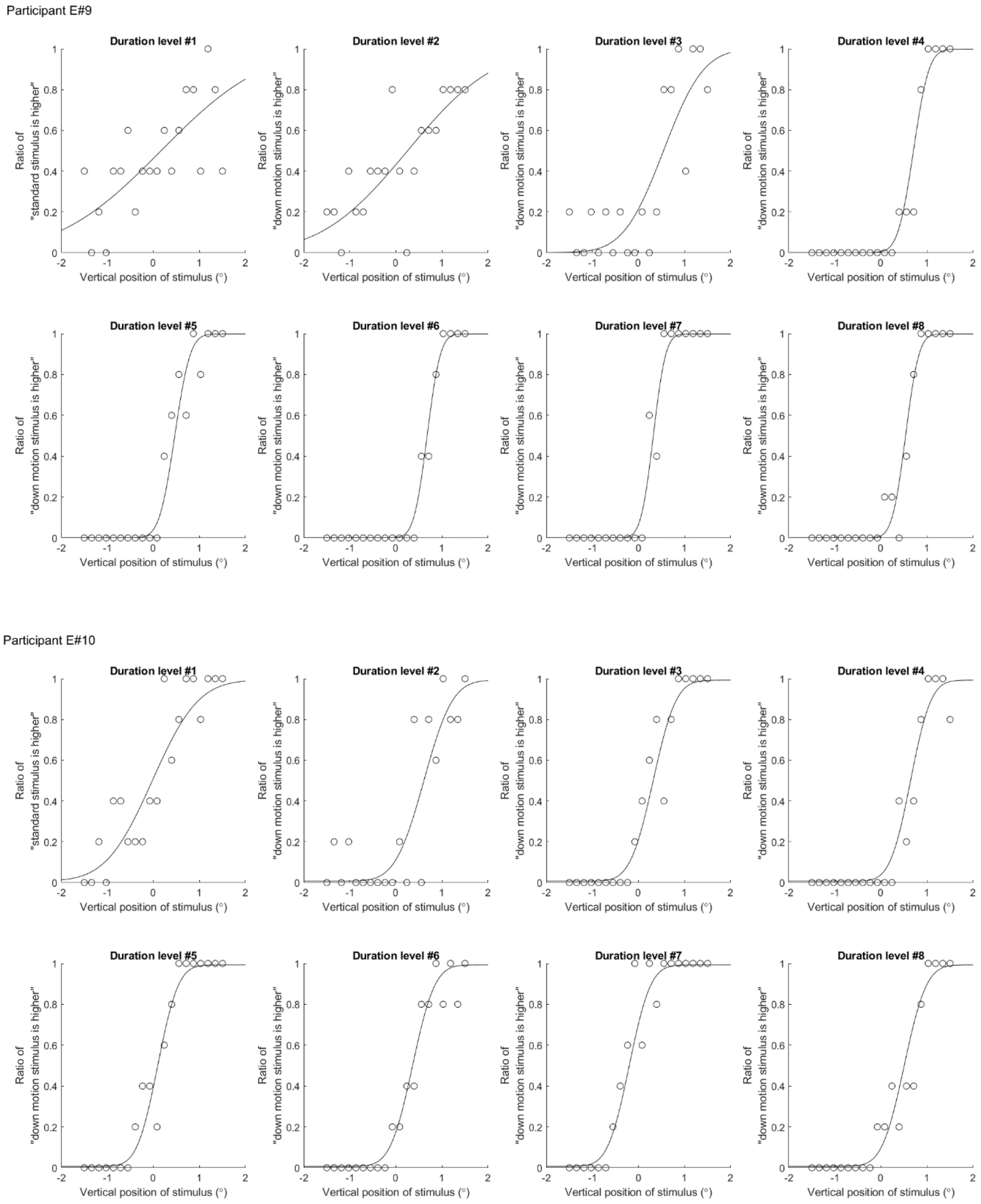

Fig. S4 (continued)

- *Young adult group*

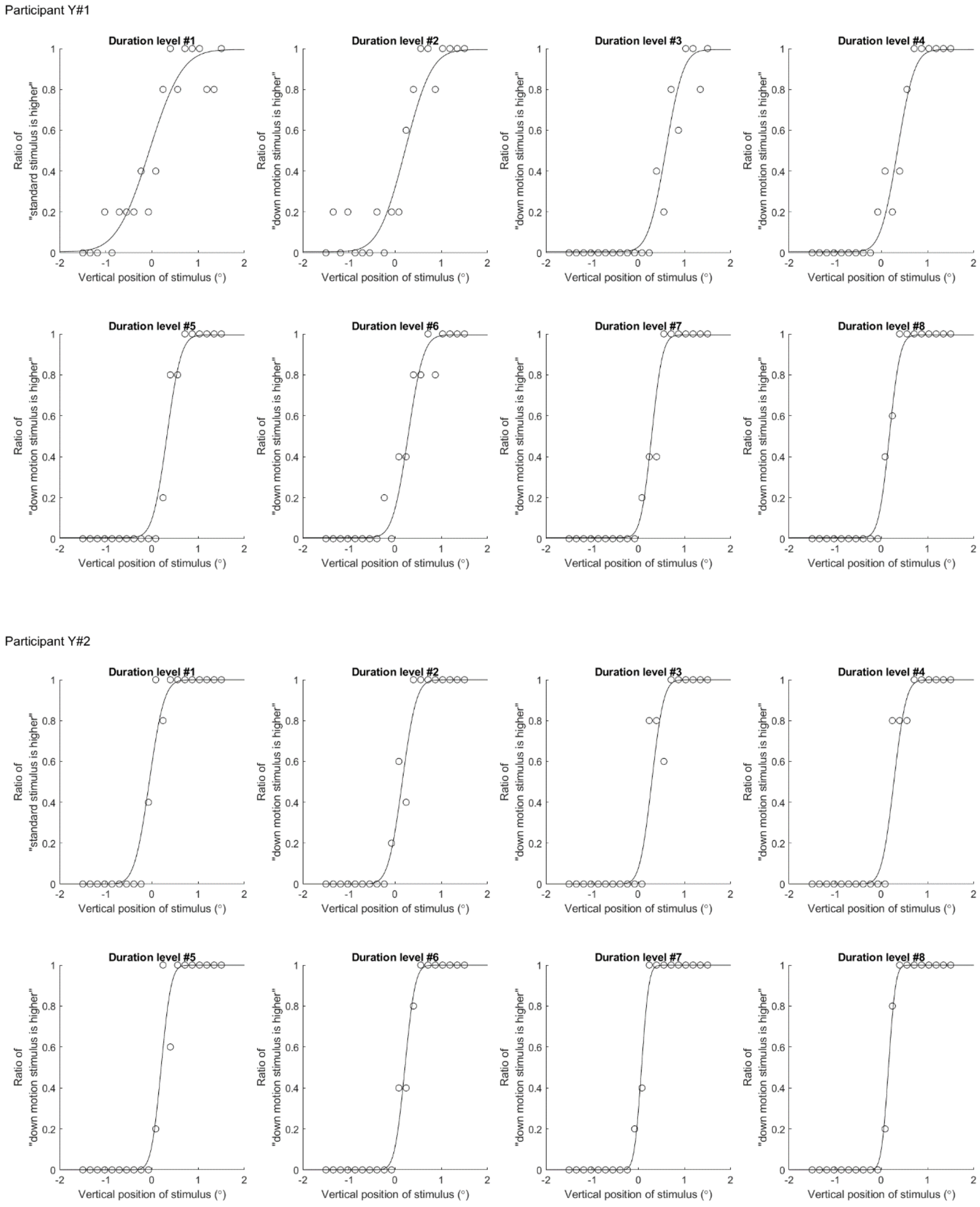

Fig. S5 (Related to Fig. 4 and Fig. 5A). Response data and fitted psychometric functions of young adult participants in Experiment 2. Dots indicate the response probability to “standard stimulus is higher” in the stationary-stimulus condition and “downward motion stimulus is higher” in the motion-stimulus condition. The standard stimulus in the stationary-stimulus condition is vertically placed as much as the vertical position of stimulus. Lines indicate fitted psychometric functions.

Participant Y#3

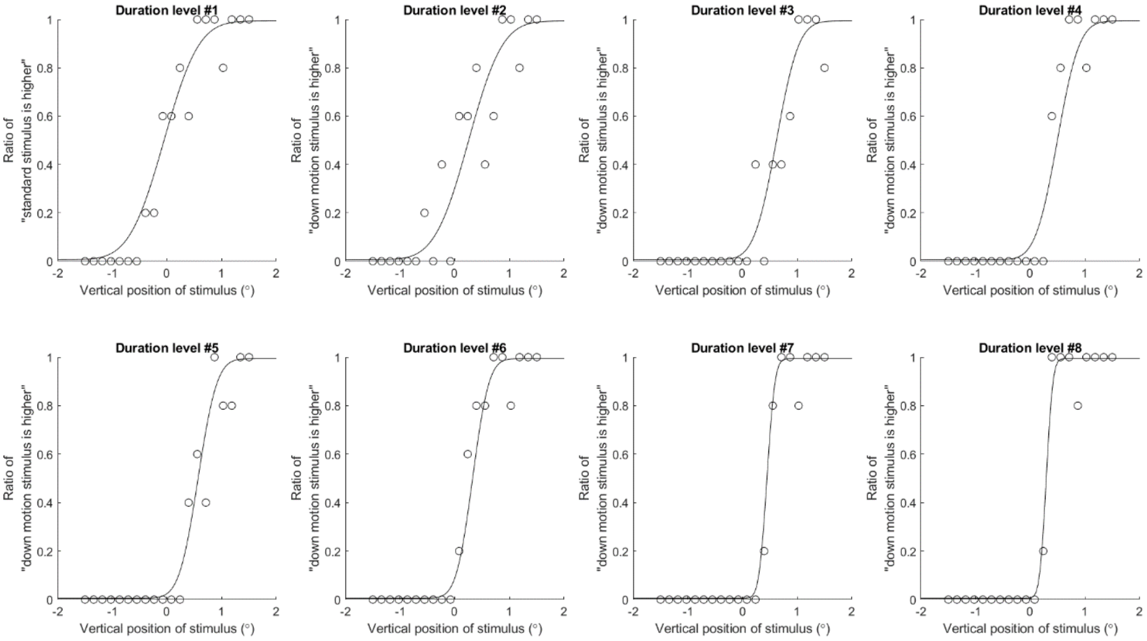

Participant Y#4

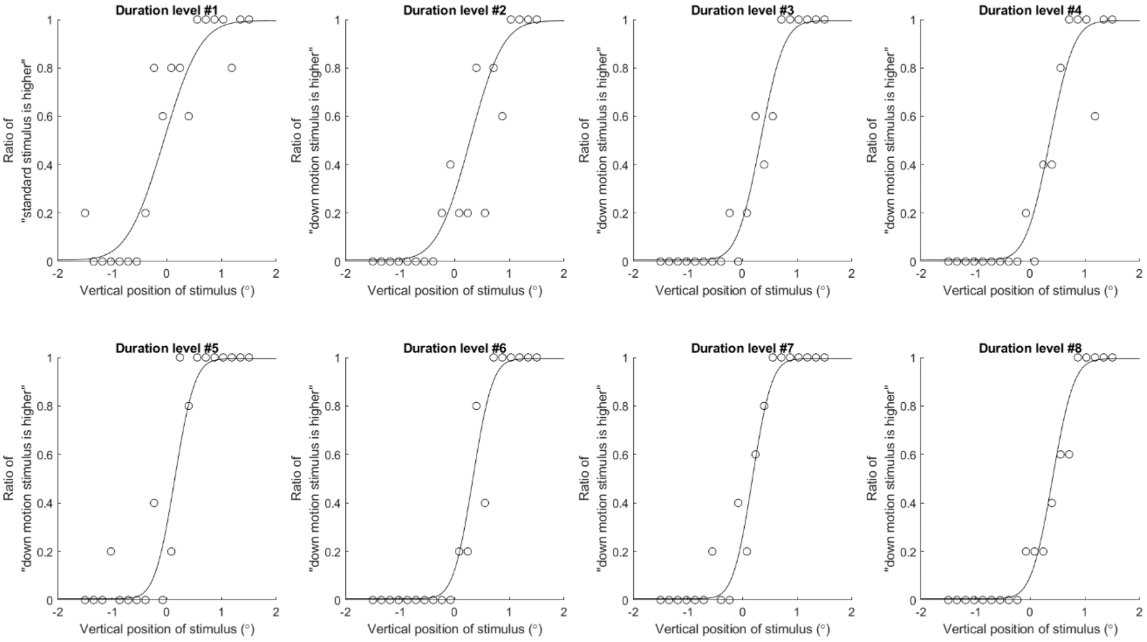

Fig. S5 (continued)

Participant Y#5

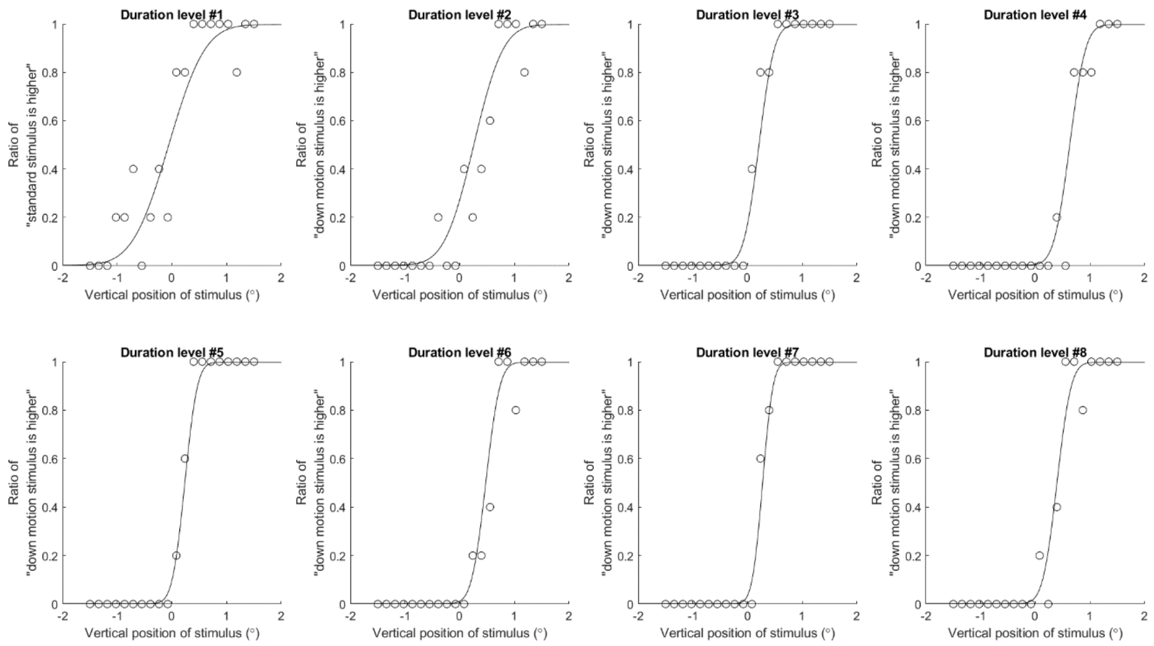

Participant Y#6

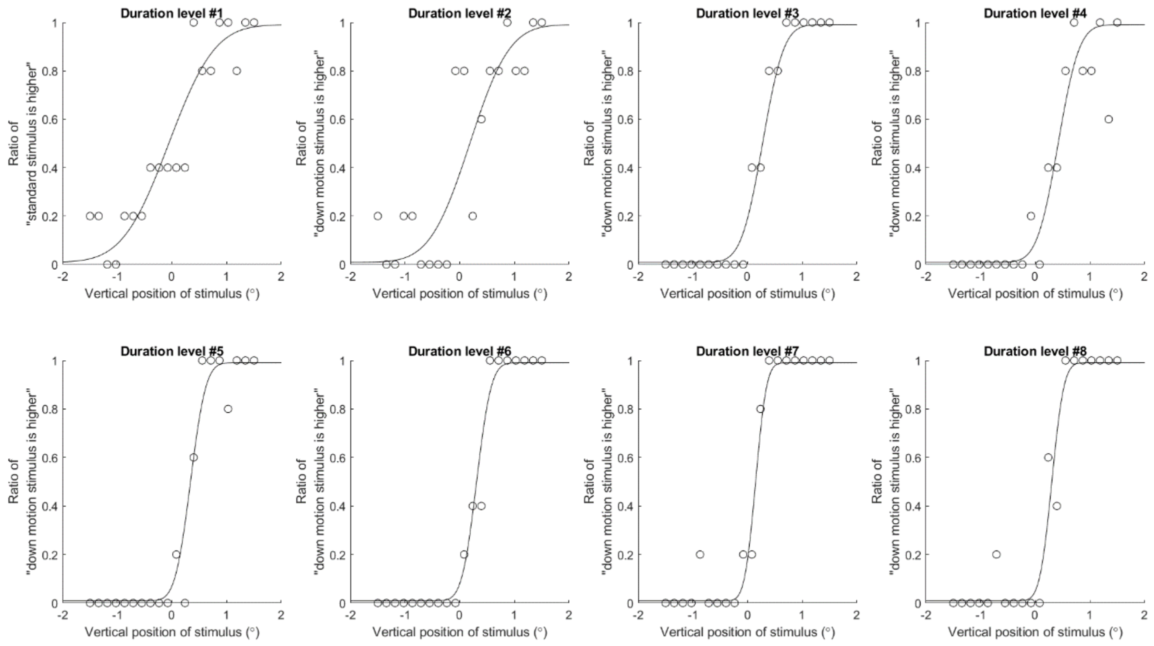

Fig. S5 (continued)

Participant Y#7

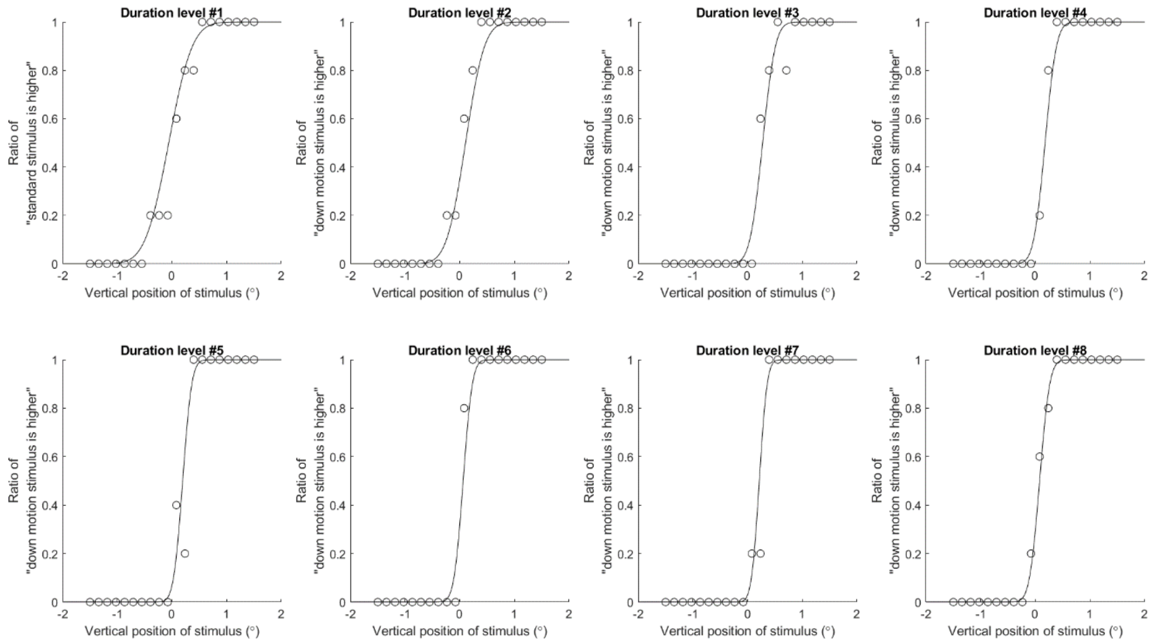

Participant Y#8

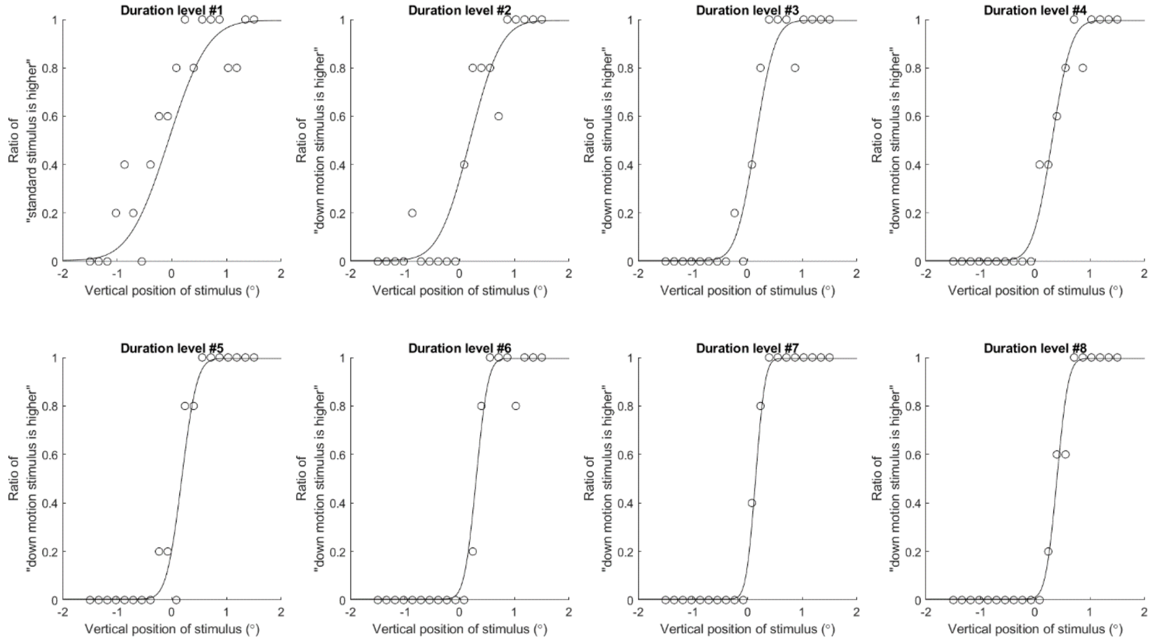

Fig. S5 (continued)
